# Ecological modeling, biogeography, and phenotypic analyses setting the tiger cats’ hyperdimensional niches reveal a new species

**DOI:** 10.1038/s41598-024-52379-8

**Published:** 2024-01-29

**Authors:** Tadeu G. de Oliveira, Lester A. Fox-Rosales, José D. Ramírez-Fernández, Juan C. Cepeda-Duque, Rebecca Zug, Catalina Sanchez-Lalinde, Marcelo J. R. Oliveira, Paulo H. D. Marinho, Alejandra Bonilla-Sánchez, Mara C. Marques, Katia Cassaro, Ricardo Moreno, Damián Rumiz, Felipe B. Peters, Josué Ortega, Gitana Cavalcanti, Michael S. Mooring, Steven R. Blankenship, Esteban Brenes-Mora, Douglas Dias, Fábio D. Mazim, Eduardo Eizirik, Jaime L. Diehl, Rosane V. Marques, Ana Carolina C. Ribeiro, Reginaldo A. Cruz, Emanuelle Pasa, Lyse P. C. Meira, Alex Pereira, Guilherme B. Ferreira, Fernando F. de Pinho, Liana M. M. Sena, Vinícius R. de Morais, Micheli Ribeiro Luiz, Vitor E. C. Moura, Marina O. Favarini, Karla P. G. Leal, Paulo G. C. Wagner, Maurício C. dos Santos, James Sanderson, Elienê P. Araújo, Flávio H. G. Rodrigues

**Affiliations:** 1 Departamento de Biologia, Universidade Estadual do Maranhão (UEMA), Campus Paulo VI, Av. Lourenço Vieira da Silva 1000, Jardim São Cristóvão, São Luís, Maranhão 65055-310 Brazil; 2 Instituto Pro-Carnívoros, Atibaia, SP Brazil; 3 Tiger Cats Conservation Initiative (TCCI), São Luís, Brazil; 4 Programa de Pós-Graduação em Ecologia, Conservação e Manejo da Vida Silvestre –ECMVS, Universidade Federal de Minas Gerais (UFMG), Belo Horizonte, MG Brazil; 5 Programa de Pós-Graduação em Ciência Animal, UEMA, São Luís, MA Brazil; 6 Oncilla Conservation, Costa Rica Wildlife Foundation, San José, Costa Rica; 7 Universidad San Francisco de Quito, Quito, Ecuador; 8 Onca Fundación para el Estudio de la Diversidad, Bogota, Colombia; 9 Instituto Biotrópicos, Diamantina, MG Brazil; 10 Universidade Federal do Rio Grande do Norte (UFRN), Natal, RN Brazil; 11 Pontifícia Universidade Católica do Rio Grande do Sul (PUCRS), Porto Alegre, RS Brazil; 12 Zoológico de São Paulo Zoo, São Paulo, SP Brazil; 13 Zoológico Beto Carrero World, Penha, SC Brazil; 14 Fundación Yaguará Panamá, Ciudad del Saber/Panama City, Panama; 15 Noel Kempff Mercado Natural History Museum, Santa Cruz de la Sierra, Bolivia; 16 Programa de Pós-Graduação em Biologia Animal, Universidade Federal do Rio Grande do Sul (UFRGS), Porto Alegre, RS Brazil; 17 Smithsonian Tropical Research Institute, Balboa Ancon, Panama; 18 GAE Serviços Ambientais, Canto do Buriti, PI Brazil; 19 Point Loma Nazarene University, San Diego, CA USA; 20 Quetzal Education & Research Center (QERC), San Gerardo de Dota, Costa Rica; 21 Re:wild, Austin, TX USA; 22 SETEG- Soluções Geológicas e Ambientais, Fortaleza, CE Brazil; 23 Ka’aguy Consultoria Ambiental, Pelotas, RS Brazil; 24 Instituto de Ciências Biológicas, Universidade Federal de Goiás (UFG), Goiânia, GO Brazil; 25 Cruzeiro do Sul Consultoria Ambiental Ltda., Ivoti, RS Brazil; 26 Bioconsultoria Ambiental Ltda., Caetité, BA Brazil; 27 Instituto Felinos do Aguaí, Siderópolis, SC Brazil; 28 Programa de Pós-Graduação em Ecologia e Conservação da Biodiversidade, UEMA, São Luís, MA Brazil; 29 Universidade Federal de Lavras (UFLA), Lavras, MG Brazil; 30 Centro de Triagem de Animais Silvestres CETAS, IBAMA-RS, Porto Alegre, RS Brazil; 31 Small Wild Cat Conservation Foundation, Corrales, NM USA; 32 Núcleo GeoAmbiental, UEMA, São Luís, MA Brazil; 33 Dept. Genética, Ecologia e Evolução, UFMG, Belo Horizonte, MG Brazil

**Keywords:** Ecology, Zoology, Ecology

## Abstract

Recently, the tiger-cat species complex was split into *Leopardus tigrinus* and *Leopardus guttulus*, along with other proposed schemes. We performed a detailed analysis integrating ecological modeling, biogeography, and phenotype of the four originally recognized subspecies—*tigrinus*, *oncilla*, *pardinoides*, *guttulus*—and presented a new multidimensional niche depiction of the species. Species distribution models used > 1400 records from museums and photographs, all checked for species accuracy. Morphological data were obtained from institutional/personal archives. Spotting patterns were established by integrating museum and photographic/camera-trap records. Principal component analysis showed three clearly distinct groups, with the Central American specimens (*oncilla*) clustering entirely within those of the Andes, namely the *pardinoides* group of the cloud forests of the southern Central-American and Andean mountain chains (clouded tiger-cat); the *tigrinus* group of the savannas of the Guiana Shield and central/northeastern Brazil (savanna tiger-cat); and the *guttulus* group in the lowland forests of the Atlantic Forest domain (Atlantic Forest tiger-cat). This scheme is supported by recent genetic analyses. All species displayed different spotting patterns, with some significant differences in body measurements/proportions. The new distribution presented alarming reductions from the historic range of − 50.4% to − 68.2%. This multidimensional approach revealed a new species of the elusive and threatened tiger-cat complex.

## Introduction

The tiger cat species complex, the original species from old taxonomic schemes, sensu Cabrera, Wozencraft, Kitchener et al.^1–3^ is one of the most intriguing, enigmatic, and fascinating group of felids. Owing to the limited knowledge available, the tiger-cat, before and after the species split^4^, has long been the subject of several preconceptions regarding where it ranges and the associated habitats. These include its presence in the Amazon Basin and in the Pantanal. The taxonomic status of the Guiana population should determine whether the recently proposed *Leopardus emiliae* would exist as a species, and even if it is mostly a forest dweller associated with montane forests, among other types of forest^5–9^. Former and recent maps of its geographic range vary considerably and even their current limits are not properly established^4–6,10–14^. Following advances in genetic studies, the group’s complexity has become ever more intriguing. Previously, when it was not known to be two different species; *tigrinus* and *guttulus* were not known to interbreed between themselves, although *L. tigrinus* from central Brazil was hybridizing with *Leopardus braccatus* (Pampas cat), and *guttulus* was exhibiting bidirectional introgression with *Leopardus geoffroyi* (Geoffroy’s cat)*.* In other words, the “same” species had no gene flow between them but interbred with two distinct species^4,15^. Meanwhile, the current *L. tigrinus oncilla* and *L. tigrinus tigrinus*, i.e., the same species, sensu Kitchener et al.^3^, comprise two completely distinct phylogenetic lines^16,17^. These discrepancies also extend to their ecological aspects, where the same species is found to have completely distinctive habitat patterns: one in the rainy cloud forests of the Andes and the other on the dry semi-arid Caatinga of Brazil^18–20^. As it stands today, the tiger cat species complex is currently composed of two species, sensu Kitchener et al.^3^—the northern tiger cat (*Leopardus tigrinus*) and the southern tiger cat (*Leopardus guttulus*), with the former is further divided into three subspecies: *L.t. oncilla, L.t. pardinoides,* and *L.t. tigrinus*.

Detailed knowledge and a proper understanding of a species’ geographic distribution and ecology are fundamental for conservation action and planning^21^. However, inadequate occurrence data for most species results in information about their distribution that is inadequate for many applications^21^. For poorly known species, determining their actual distribution is a major first step toward establishing management action. Beyond determining the range of the species, determining the patches of suitable habitat within that range is also crucial.

The n-dimensional hypervolume occupied by a species, a concept invoked by Hutchinson^22^ to describe the ecological niche of any living being, has been applied predominantly to depict hypervolume geometry, morphological hypervolumes, or climate hypervolumes^23^, but not used in an integrative manner.

Ecological niche modeling has become a useful tool for delineating species distributions^24–26^, identifying conservation priorities^27,28^, and even predicting range shifts under different climate change scenarios^29,30^. Niche models have also been used to support the recognition of cryptic species or for elevating populations and subspecies to species status^31,32^. The latter has important implications, as threat assessments often take into account only the whole species at the global level rather than populations or subspecies^33^.

*L. tigrinus* and *L. guttulus* are both currently defined as globally threatened species^12,13^. They inhabit some of the most threatened ecoregions and biodiversity hotspots in the Americas, including the Cerrado, Tropical Andes, Atlantic Forest, and the Talamanca mountain range. With the exception of *L. tigrinus tigrinus*, there are no published conservation priority areas for the entities in the complex. Given their threatened status and the high rates of habitat loss within their respective ranges, it is imperative to identify the areas that are more likely to harbor viable populations of these species.

In light of all these issues, we sought to determine the actual distribution range of the tiger-cat species, compare the ecological and biogeographic characteristics of different species/subspecies, and assess the similarities and differences in morphology and phenotype among them. Ultimately, we aimed to characterize the tiger-cats phenotypically, biogeographically, and ecologically to determine the actual number of species. For this, we used a combination of ecological niche modeling and phenotypic analyses (morphologic and skin characteristics), to differentiate the ecological niche of the tiger-cat species complex. We have compiled the largest dataset of occurrence ever for the complex and used species distribution models to define the range and distribution limit of each species of the complex. We also identify the climatic and land cover factors that exert the greatest influence on their respective distributions and the historic decline in species range. We also identified conservation priority areas for each species. Further, our results reveal the existence of a cryptic species, the clouded tiger-cat (*L. pardinoides*), which includes both *L.t. oncilla* and *L.t. pardinoides*. The split of the complex into three species highlights the urgent need for updated threat assessments for each of the individual species.

## Results

### Species distributions, biogeographic issues, and ecological niche modeling

In total, we gathered 1439 records: 494 for *L. guttulus*, 114 for *L.t. oncilla*, 237 for *L.t. pardinoides,* and 594 for *L.t. tigrinus*. This number of data points is unprecedented in terms of quantity and breadth of range area covered (Supplementary Fig. S1); these records completely reshape the current known distribution adopted by the IUCN^12^. Only 12% of our records came from museum specimens; the vast majority (88%) was from living animals (mainly photographic material, with a few roadkills).

**Figure 1:**
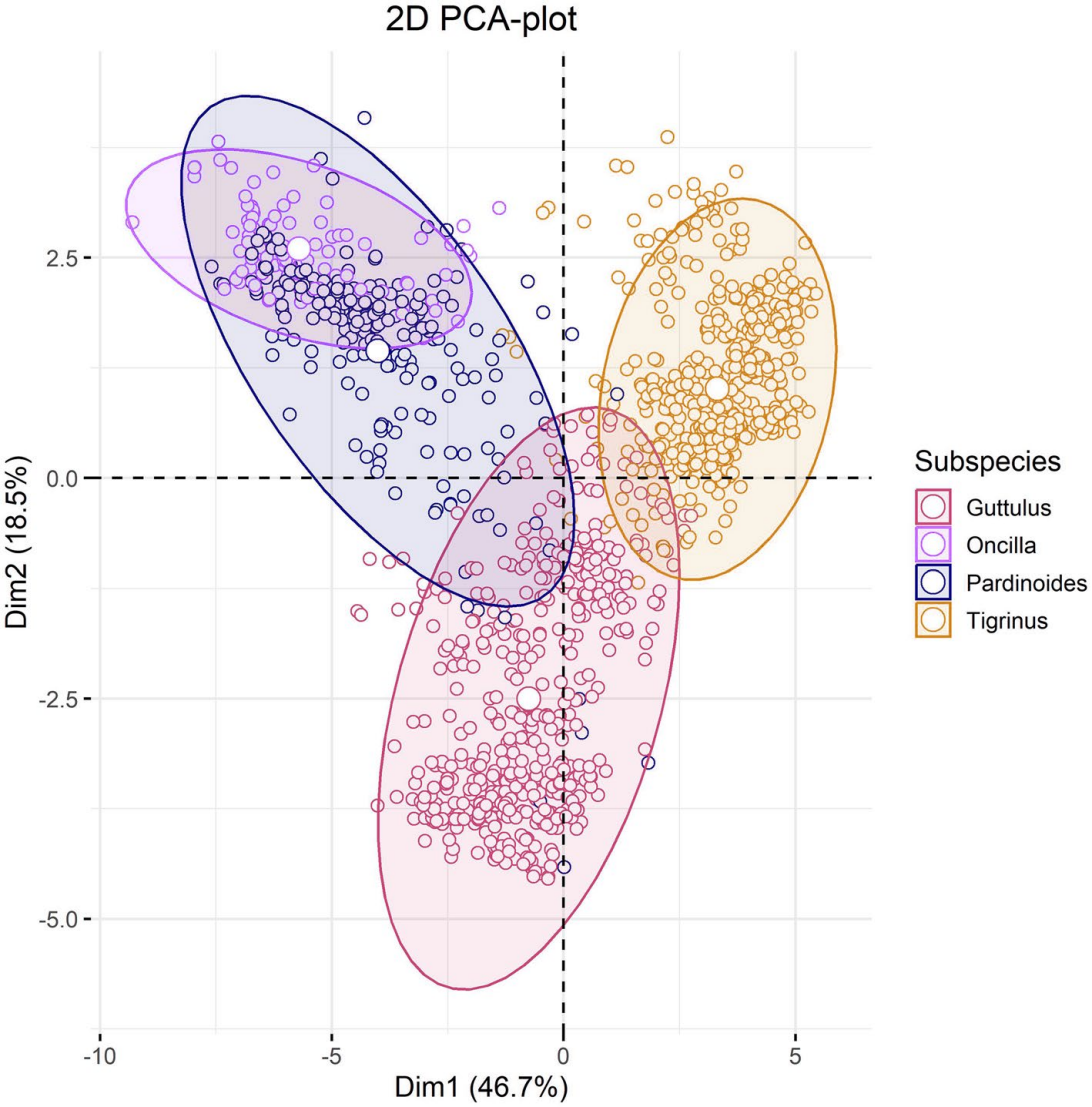
Principal component analysis (PCA) of the tiger cat species complex reveals three groupings, setting the species apart, with the *oncilla* completely within *pardinoides*, the *pardinoides* group (clouded tiger-cat), the *tigrinus* group (savanna tiger-cat), and the *guttulus* group (Atlantic Forest tiger-cat).

**Figure 2:**
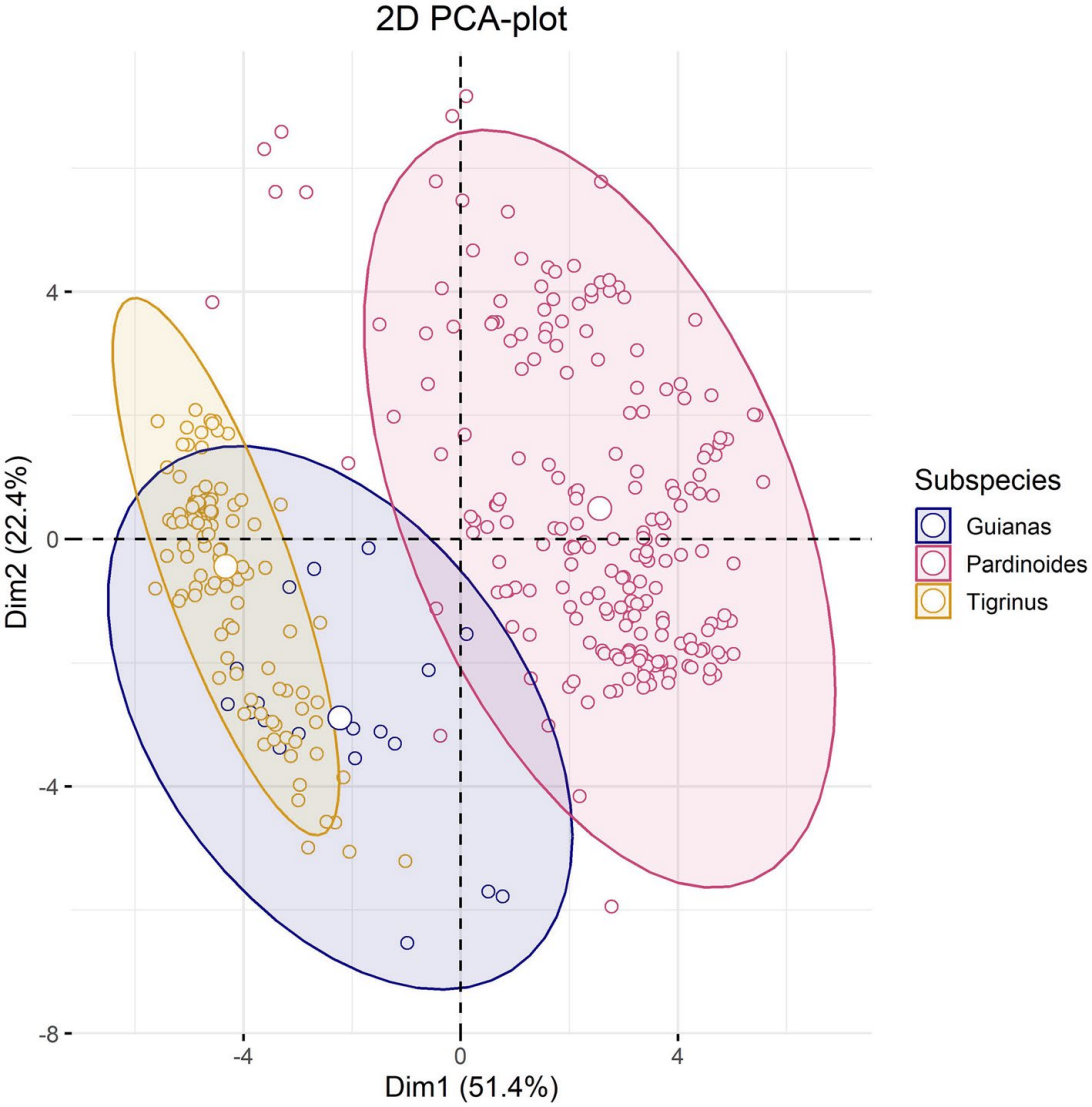
Principal component analysis of savanna tiger-cats from the Guiana Shield, with those of eastern Amazonia and the northern savannas, and with clouded tiger-cats (*L. pardinoides*). PCA clearly shows convergence between the two groups of *L. tigrinus*, and not with those of *L. pardinoides*; such a scheme would invalidate *L. emiliae* as a species in as far as ecological niche dimensions are concerned.

The Principal component analysis (PCA) plot of the entire tiger-cat species complex—the original *Leopardus tigrinus/Felis tigrina* (Schreber, 1775^34^), sensu Cabrera, Wozencraft^1,2^ —showed marked separation among *tigrinus*, *pardinoides*, and *guttulus,* but a complete overlap between the original *pardinoides* and *oncilla* subspecies (Fig. 1). Phenotypically, the latter two are also remarkably alike; henceforth, we grouped them together for further analysis under “*pardinoides*” and considered the Principal component analysis (PCA) groupings as three distinct species. A PCA side analysis showed that the Guianas’ specimens grouped with *tigrinus*, but not *pardinoides* (Fig. 2). MANOVA also stressed the marked differentiation among the three tiger-cat species for all 25 niche variables (Pillai’s Trace = 1.7, *F*_2, 1436_ = 322.85, *P* = 2.2 × 10^−16^), which was confirmed by individual ANOVAs for each parameter (*P* < 0.001; Supplementary Table S1).

To model species distribution, we applied a 3 km filter, which led us to use 1051 records for modeling (435 *L. tigrinus*, 211 *L. pardinoides*, and 405 *L. guttulus*). The species distribution models performed well for all three species: *L. pardinoides* (AUC = 0.97, omission = 0.02, *P* < 0.001), *L. tigrinus* (AUC = 0.93, omission = 0.13, *P* < 0.001), and *L. guttulus* (AUC = 0.93, omission rate = 0.07, *P* < 0.001).

The variables with the greatest influence on *L. pardinoides* distribution were annual mean temperature (bio1, 28.1%), terrain ruggedness (TRI, 25.2%), and ecoregion (18.4%). The effect of annual mean temperature was negative, with suitability rapidly declining at values > 17 °C. Terrain ruggedness had a positive effect on suitability, with high habitat suitability at values > 500 m. These results characterize the species as a cloud forest/tropical highland specialist.

For *L. tigrinus*, the most influential variables were land cover (53.8%), tree cover (15.1%), and ecoregion (5.9%). In terms of land cover, the species strongly preferred shrublands; there was medium to low suitability in tree cover areas and no suitability in all other land cover types. Suitability for the species increased in areas with tree cover values of 0–35%, but declined at values above 35%. In terms of ecoregion, the two with the highest suitability were the Caatinga and Cerrado domains.

Finally, *L. guttulus* distribution was mostly influenced by land cover (57.6%), the maximum temperature of warmest month (bio5, 8.7%), and gross primary production (GPP, 7.5%). In terms of land cover, the species strongly preferred tree cover areas; there was medium to low suitability in shrubland areas and no suitability for all other land cover types. Suitability with regard to maximum temperature of the warmest month was highest between 19 and 24 °C, but declined rapidly above 24 °C, suggesting that the species range to the north and northeast is limited by the hotter and arid climates characteristic of the Caatinga and Cerrado domains. Finally, with regards to GPP, suitability increased as GPP increased. Although low values of GPP also resulted in high suitability, this may be artifactual owing to several records found along the coast, where the GPP layer did not project well.

**Figure 3:**
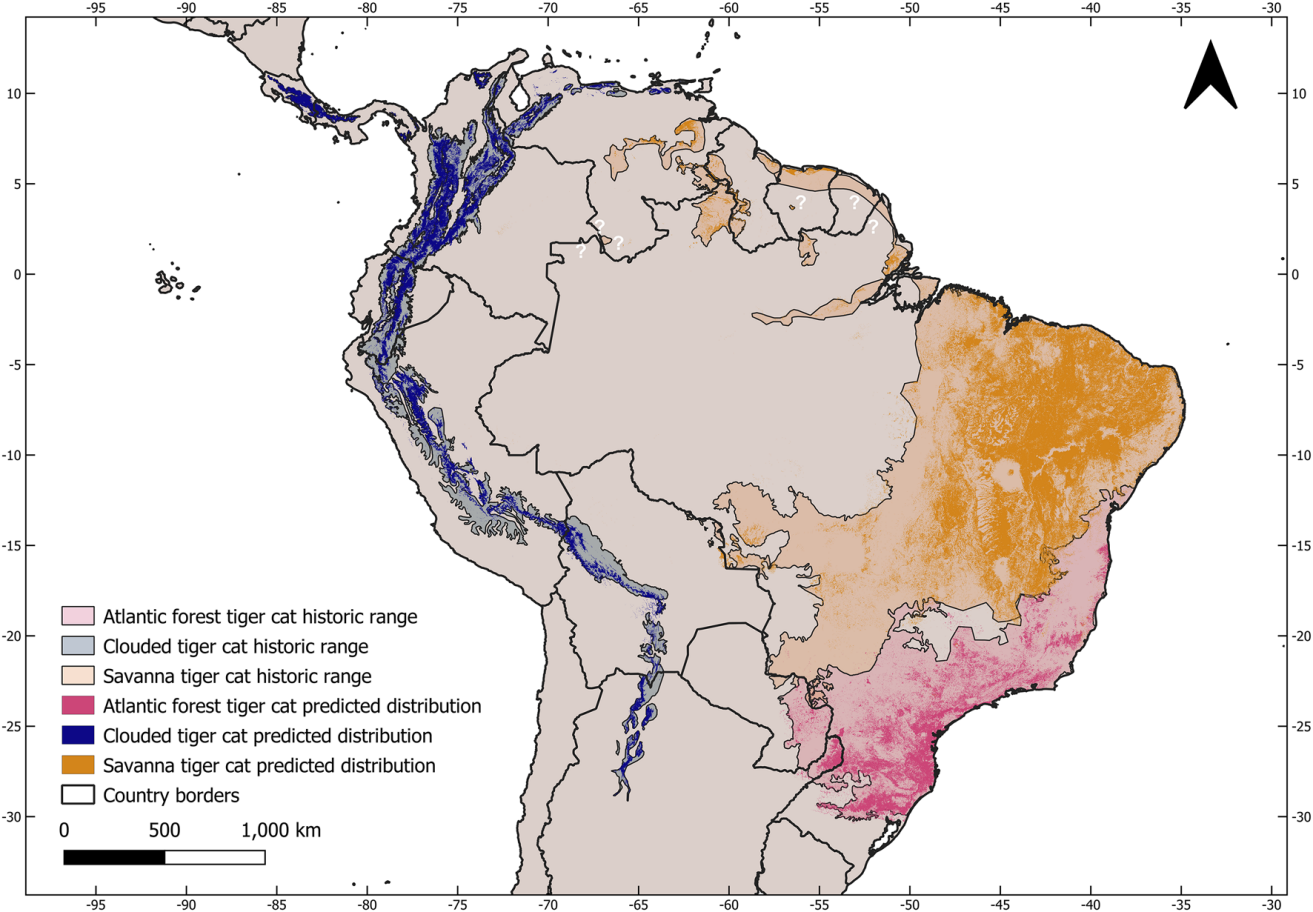
Historic and current distribution of the tiger-cat species complex: savanna tiger-cat (*Leopardus tigrinus*), Atlantic Forest tiger-cat (*Leopardus guttulus*), and the new species, the clouded tiger-cat (*Leopardus pardinoides*). Figure made on QGIS v. 3.28.12 LTR (www.qgis.org).

The likely historic range of the three tiger cat species totaled 5,481,237 km^2^, which is only 47.5% of their current 11,535,141 km^2^ reported by the IUCN (Table 1). Our current and historic range (Fig. 3) shows considerable area reductions for all species of approximately 50–70%. Considering the current IUCN range for *L. guttulus* and *L. tigrinus* (which includes *tigrinus, pardinoides*, and *oncilla, *sensu Kitchener et al.^3^ and our current model, the area reduction would be a massive 80%. These red alarm-setting reductions will have considerable impacts on the conservation assessment and other issues for all the tiger-cat species concerned, although each has its particularities.

**Table 1:** Historic and current range (in km^2^) of the tiger-cat species, their area loss, and comparisons with the current IUCN range map and the reduction percentages in relation to the current ranges.

Species	Historic range (km^2^)	Current range (km^2^)	Reduction (%)	IUCN range 2016 (km^2^)	Reduction (%)
*L. tigrinus*	3,400,771	1,498,860	− 55.9	9,389,255	− 79.6
*L. pardinoides*	824,923	409,371	− 50.4		
*L. guttulus*	1,255,543	399,604	− 68.2	2,145,887	− 81.4

**Figure 4:**
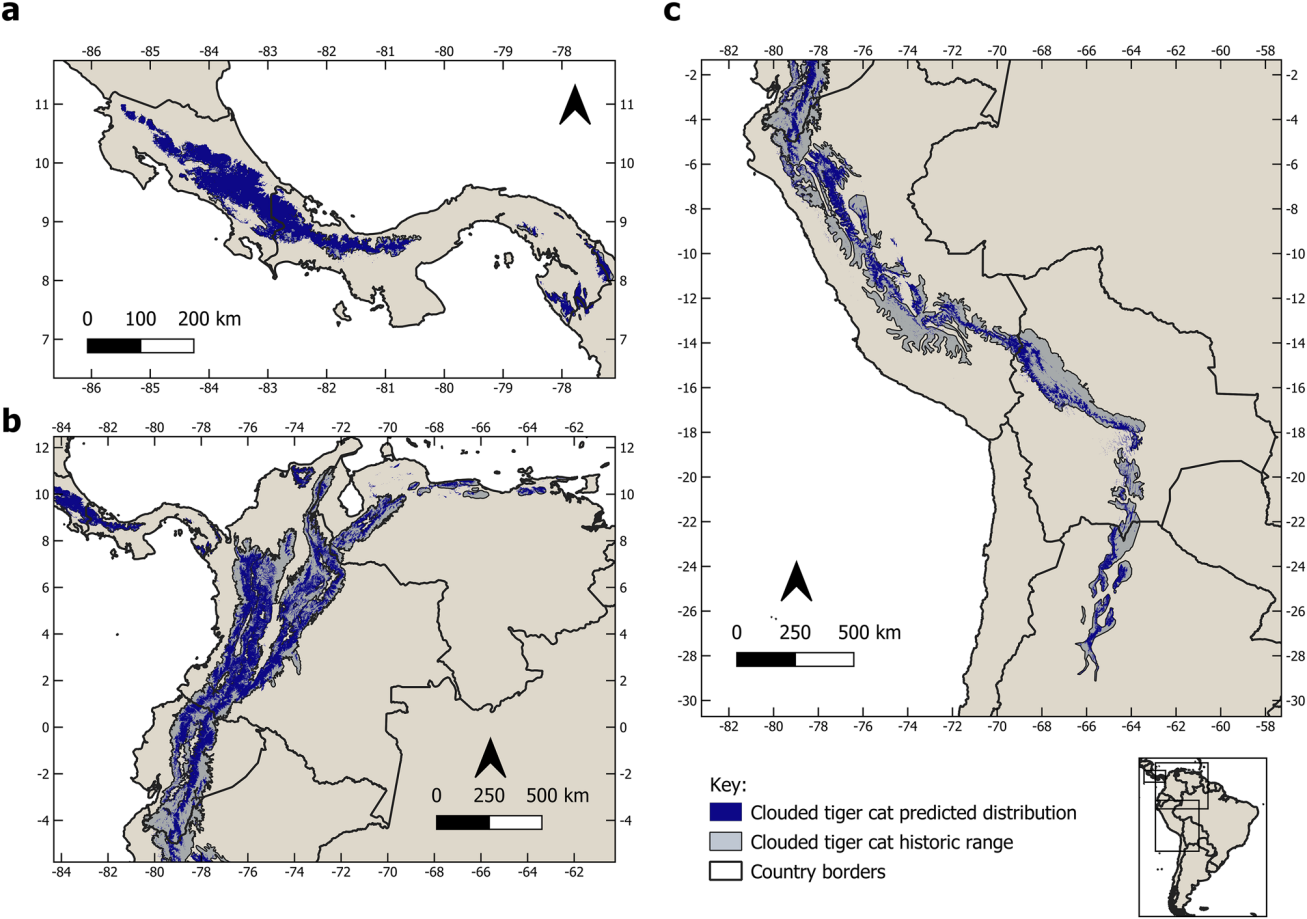
Current and historic range of the clouded tiger-cat (*Leopardus pardionides*) in Central America (**a**), the northern Andes (**b**), and the southern Andes (**c**). Figure made on QGIS v. 3.28.12 LTR (www.qgis.org).

The distribution of the entire complex is very biome oriented. The clouded tiger-cat, *L. pardinoides*, ranges along 11 mountain ecoregions. In Central America, it is restricted to the Tilarán, Central Volcanic, and Talamanca cordilleras of Costa Rica and Panama (the Talamancan montane forests ecoregion) and the eastern Panamanian montane forests. The lowland rainforest and swamps of the Atrato river basin in the Chocó–Darién ecoregion is the primary barrier between the eastern Panamanian and Andean populations of clouded tiger-cat, while to the north, the species is limited by the Isthmian–Atlantic rainforest. In South America, the range extends from the Venezuelan Andean forests through the eastern, central, and western cordilleras of Colombia, into Ecuador, all the way through the Peruvian, Bolivian, and Southern Andean Yungas forests ecoregions, and ends in northwest Argentina (Fig. 4). The core area of its distribution lies in Colombia, but extends into Ecuador. Thus, it becomes clear that *L. pardinoides* is the tiger-cat species of the cloud forests of the southern Central American and Andean mountain chains.

**Figure 5:**
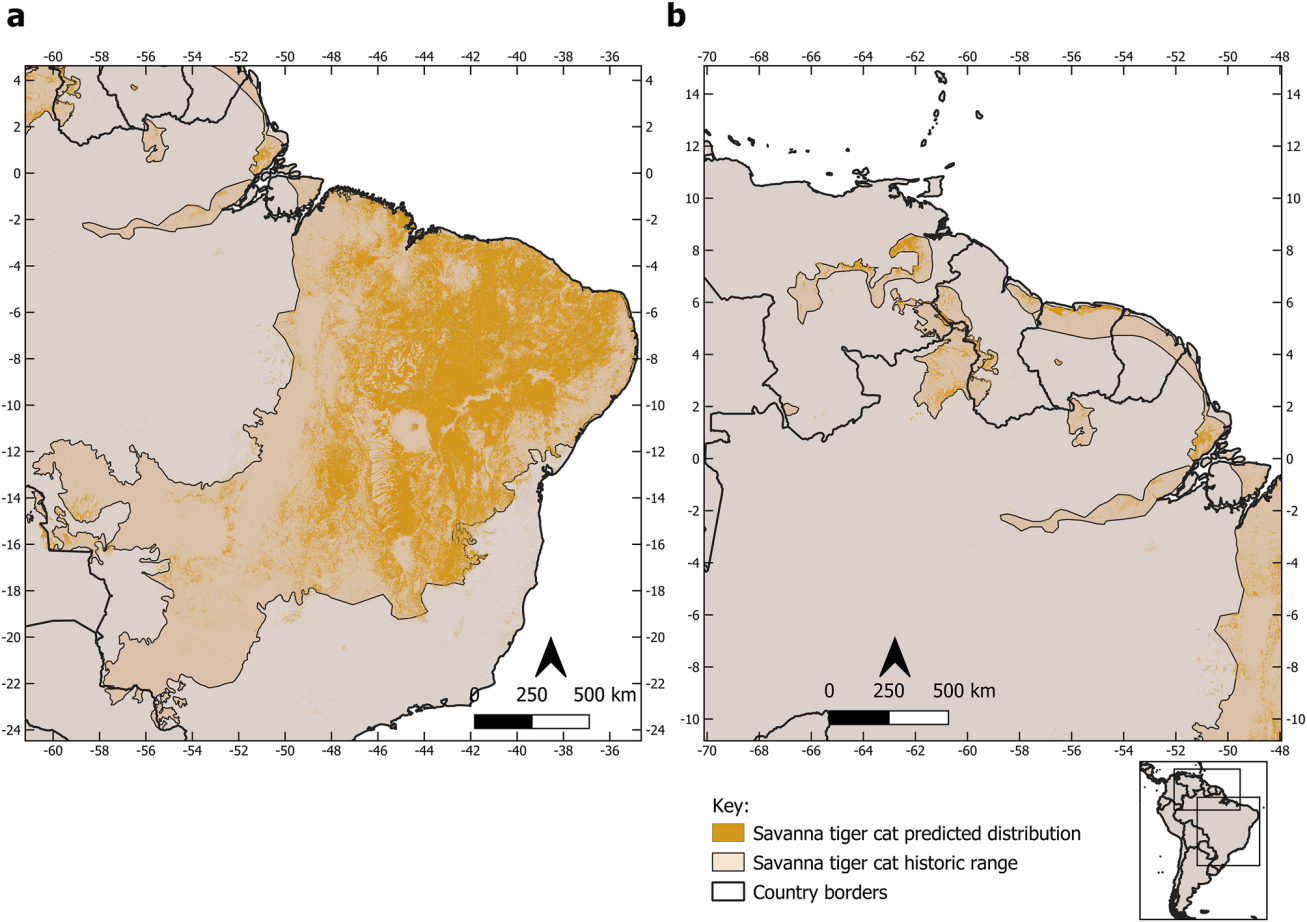
Current and historic range of the savanna tiger-cat (*Leopardus tigrinus*) in northern/northeastern/central Brazil (**a**) and the Guiana Shield (**b**). Figure made on QGIS v. 3.28.12 LTR (www.qgis.org).

The range of the savanna tiger-cat, *L. tigrinus*, is restricted to the savannas and part of the forests of the Guiana Shield, through eastern Amazonia (west of the Tocantins River), the savannas of the Cerrado biome, and the semi-arid shrub-woodlands of the Brazilian Caatinga (Fig. 5). The core of this species distribution lies in the northern MATOPIBA (an acronym for the states of Maranhão, Tocantins, Piauí, and Bahia) savannas and the Caatinga shrub-woodlands of northeastern Brazil. Thus, *L. tigrinus* is the species of the savanna formations.

**Figure 6:**
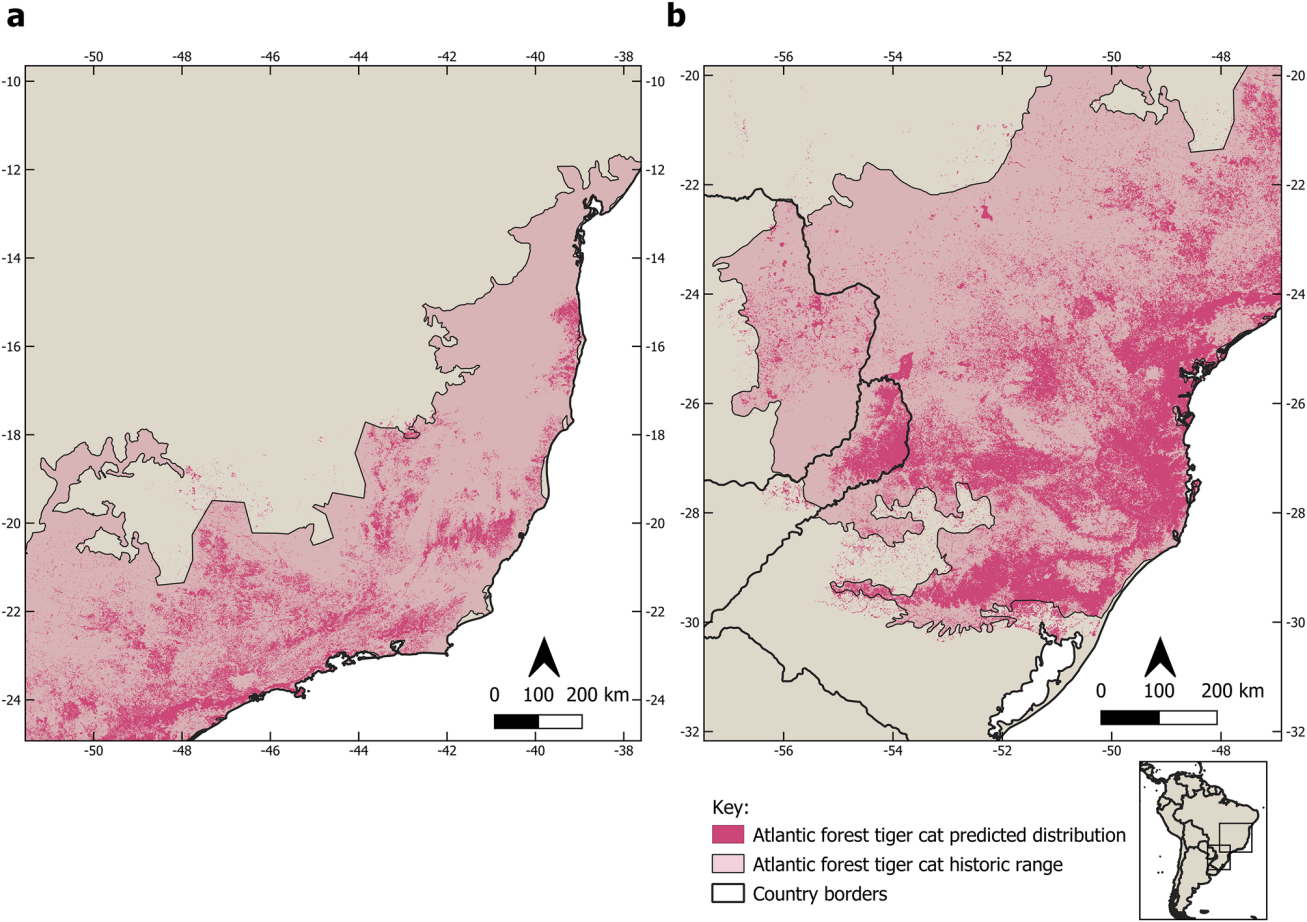
Current and historic range of the Atlantic Forest tiger-cat (*Leopardus guttulus*) in the northern (**a**) and southern (**b**) part of its range. Figure made on QGIS v. 3.28.12 LTR (www.qgis.org).

Among all the tiger-cats, the Atlantic Forest tiger-cat (*L. guttulus*) is essentially restricted to the ecoregion of the Atlantic Forest domain of Brazil, which extends from the coastal area of southeast Bahia into Paraguay and the Misiones region of northeast Argentina to the Central Depression of Rio Grande do Sul State in Brazil, beyond which lies the Pampa formations (Fig. 6). Currently, the core distribution of this species lies in the fertile soils of southern Brazil, especially in the states of Paraná and Santa Catarina. Outside of this area, its distribution appears much patchier owing to deforestation.

The range of the clouded tiger-cat is completely isolated from the other species, mostly through lowland rainforests, such as those of the Isthmian–Atlantic, Chocó–Darién, and the Amazon. Although the savanna and Atlantic Forest tiger-cats do not seem to overlap, our records and modeling showed that there could be narrow contact zones between them in the border area of these biomes in southeast Bahia and northeast Minas Gerais, which would be facilitated by deforestation.

Regarding countries, three emerge as critical for the species complex: Costa Rica for the *L. pardinoides oncilla* population (60% of the subspecies range), Colombia for *L. pardinoides* (39.6% of the total species range), and Brazil for both *L. tigrinus* (98% of the total species range) and *L. guttulus* (92.9% of the total species range) (Supplementary Table S2).

### Setting the species apart

At a first glance, all tiger-cats may appear the same, especially for those without experience of the species. However, some characteristics are very distinctive and allow their differentiation. Their morphological body measurements and proportions show subtle differences that are also found in some phenotypical features (Table 2). A detailed phenotypic characterization is presented in the Supplementary Material: Characterizing the Species, Diagnostic Differences from other Sympatric *Leopardus*, Taxonomic Notes.

**Table 2:** Comparison of the multidimensional hypervolume niches for the tiger-cat species, presenting the body measurements (mean and range, more details in Table S3) and diagnostic features of clouded (*L. pardinoides*), savanna (*L. tigrinus*), and Atlantic Forest (*L. guttulus*) tiger-cats: their multidimensional hypervolume niche depicted.

	*L. pardinoides*	*L. tigrinus*	*L. guttulus*	References
Body mass (kg)	2.27 (1.8–3.4)	2.32 (1.85–3.46)	2.38 (1.7–3.47)	
Head and body (HB) (mm)	485 (422–540)	482 (380–650)	510 (460–591)	
Tail (T) (mm)	290 (245–340)	278 (210–345)	272 (212–310)	
T/HB%	60 (52–69)	58 (50–67)	53 (43–59)	
% w/long margay-sized tail	71.4	65	31	
% w/average tail	28.6	35	69	
Ear (mm)	38.6 (30–45)	42.9 (36–50)	41.6 (30–51)	
E/HB%	7.8 (5.6–8.7)	9.1 (6.5–10.9)	8.1 (6.4–10.3)	
Pairs of mammae	1	2	2	
Upper canine diameter (mm)	4.24	4.06	4.05	
Body pattern	Long margay-sized bushy tail, short-round ears, margayish-looking head	Slender long-legged body, long margay-sized thin tail, proportionally large ears	Slightly bulky body, medium-large bushy tail, proportionally smaller ears	
Overall ground color	Rich reddish/orangish/grayish-yellow	Yellowish, grayish-yellow	Reddish/brownish-yellow tone	
Spot patterns: side	Irregularly shaped medium-large “cloudy” rosettes, strongly marked that often times coalesce	Small open or small/large solid dot-like rosettes that may coalesce or tend to coalesce	Large and numerous round rosettes, very rarely tending to coalesce	
Geographic range	Southern Central-American mountains, Andean chain to NW-Argentina	Guiana Shield, E-Amazonia, NE/Central/NW-Brazil	SE-Bahia, SE/S-Brazil to E-Paraguay, and NE-Argentina	
Elevation (meters above sea level [asl])	Mostly > 1500 m, especially 2000–3000 m, up to 3960 m	Mostly lowland, up to 1254 m	Mostly lowland up to 1817 m	
Soil fertility (net primary production in kgC/m^2^)	Fertile soils (10,773)	Mostly in less productive soils (5810)	Fertile soils (13,937)	
Temperature	Mild	Hot	Warm	
Climate	Sub-tropical/temperate climate with very abundant rainfall	Tropical dry/semi-arid	Tropical/sub-tropical areas with either hot or mild summers with high rainfall	
Tree cover/canopy height	High tree cover (90%), canopy height of 20 m	Low tree cover (typically < 50%), median canopy height of 3 m	Tree cover of 89% (high variation: 0–100%), canopy height of 12 m	
Main habitat type	Cloud forest	Savanna and shrub woodland formations with dense underbrush	Forests	
Interspecific relations with the dominant mesopredator, “ocelot effect”	Population/habits negatively affected by medium/high ocelot numbers (not at low levels)	Population/habits negatively affected by medium/high ocelot numbers (not at low levels)	Population/habits negatively affected by medium/high ocelot numbers (not at low levels)	^70,71^
Interspecific relations with other small felids	Population/habits not affected by other sympatric small-sized felids	Shows habitat and temporal segregation from *L. braccatus* but no pop. effect; population/habits not affected by other sympatric small-sized felids	Range segregation with *L. geoffroyi*; population/habits not affected by other sympatric small-sized felids	^65,71,92^; this work
Red List status assessment (2016)	Vulnerable (included in *L. tigrinus*)	Vulnerable (including *L. pardinoides*)	Vulnerable	^12,13^
Occupancy negatively affected by proximity with human settlements?	Yes	Yes	Yes	^18,20,74^
Area reduction from historic range (%)	− 50.4	− 55.9	− 68.2	
Autosomal heterozygosity	Average	Very low	Average	^69^
Introgression with other species	None known	Mitochondrial DNA of *L. braccatus*	Bidirectional with *L. geoffroyi* in their contact zone	^4^
Phylogenetic split from nearest ancestral line (million years ago [MYA])	2.45 MYA	1.52 MYA	1.52 MYA	^16,17^

#### Morphological measurements

There was considerable overlap in the range of body measurements of *L. tigrinus, L. pardinoides*, and *L. guttulus* (Table 2). Parameters such as body mass (*H* = 0.960, *df* = 2, *P* = 0.619) and ear size (*F*_2, 48_ = 1.886, *P* = 0.163) were not different. However, upon closer inspection, we observed significant differences in head and body length (HB) (*H* = 11.466, *df* = 2, *P* = 0.003). *L. guttulus* (510 mm) had a larger HB than *L. tigrinus* (482 mm; *T* = 484.5, *P* = 0.003) and *L. pardinoides* (485 mm; *t* = 2.828, *df* = 63, *P* = 0.006), which did not differ in size (*T* = 485.5, *P* = 0.399). Tail size was also different among all species (*F*_2, 83_ = 3.685, *P* = 0.029): that of *L. pardinoides* (290 mm) was significantly larger than that of *L. guttulus* (272 mm; *t* = 2.936, *df* = 64, *P* = 0.005), but not different from *L. tigrinus* (278 mm; *t* = 1.308, *df* = 39, *P* = 0.198), which does have a longer tail (see Table 2, Supplementary Table S3), but it is not significantly different from *L. guttulus* (*t* = 0.931, *df* = 63, *P* = 0.355). However, when we compare the T/HB proportions, considerable changes can be observed. To assess the long tail pattern exhibited by several individuals of the complex, we compared their tail proportions with those of the margay, which has 56–79% of HB, to evaluate the proportion of margay tail-sized tiger cat specimens of all groups (i.e., tail ≥ 0.56 of HB). Only approximately 31% of *L. guttulus* had a large margay-sized tail; the vast majority (69%) presented the standard medium-large tail characteristic of the species. Conversely, most *L. tigrinus* and *L. pardinoides* had long margay-sized tails (65% and 71.4%, respectively); only 35% and 28.6% displayed the medium-large pattern characteristic of *L. guttulus*. Thus, *L. tigrinus* had a significantly proportionally larger tail than *L. guttulus* (*t* = 3.806, *df* = 62, *P* < 0.001), a difference that was not observed when comparing only their total tail measurement. In addition to *L. tigrinus*, *L. pardinoides* had proportionally larger, margay-sized tails compared with their HB length than *L. guttulus* (*t* = 5.705, *df* = 63, *P* < 0.001), where they are medium-large; however, there was no difference between T/HB in the former two (*t* =  − 1.279, *df* = 39, P = 0.208), just as for total tail size.

Ears were proportionally (E/HB) larger (by 1 cm) (*F*_2, 46_ = 5.301, *P* = 0.008) in *L. tigrinus* than in *L. pardinoides* and *L. guttulus* (*t* = 2.777, *df* = 25, *P* = 0.010; *t* = 2.678, *df* = 37, *P* = 0.011, respectively), but did not differ between the latter two (*t* =  − 0.837, *df* = 30, *P* = 0.409). Thus, we could say that the savanna tiger cat has bigger ears. Meanwhile, as shown before, the ear size measurement by itself was not different between all three species.

The canine diameter (Supplementary Table S3) did not differ within the tiger cat complex (*F* = 0.404, *P* = 0.67). Thus, from the comparison of the canine width of the entire species complex (4.08 ± 0.41 mm, *n* = 78) with that of the margay (5.24 ± 0.64 mm, *n* = 76), the former had a significantly smaller diameter (*T* = 8549.5, *n* = 76, 78, *P* < 0.001). There was, however, a significant difference in the length of the upper carnassial: 9.98 ± 0.52 mm (range 9–10.85 mm, *n* = 37) in the tiger cat complex and 11.14 ± 0.77 mm (range 9.6–12.61 mm, *n* = 62) in the margay (*t* =  − 8.117, *df* = 97, *P* < 0.001).

#### Niche dimensions of the tiger cat species complex

**Figure 7:**
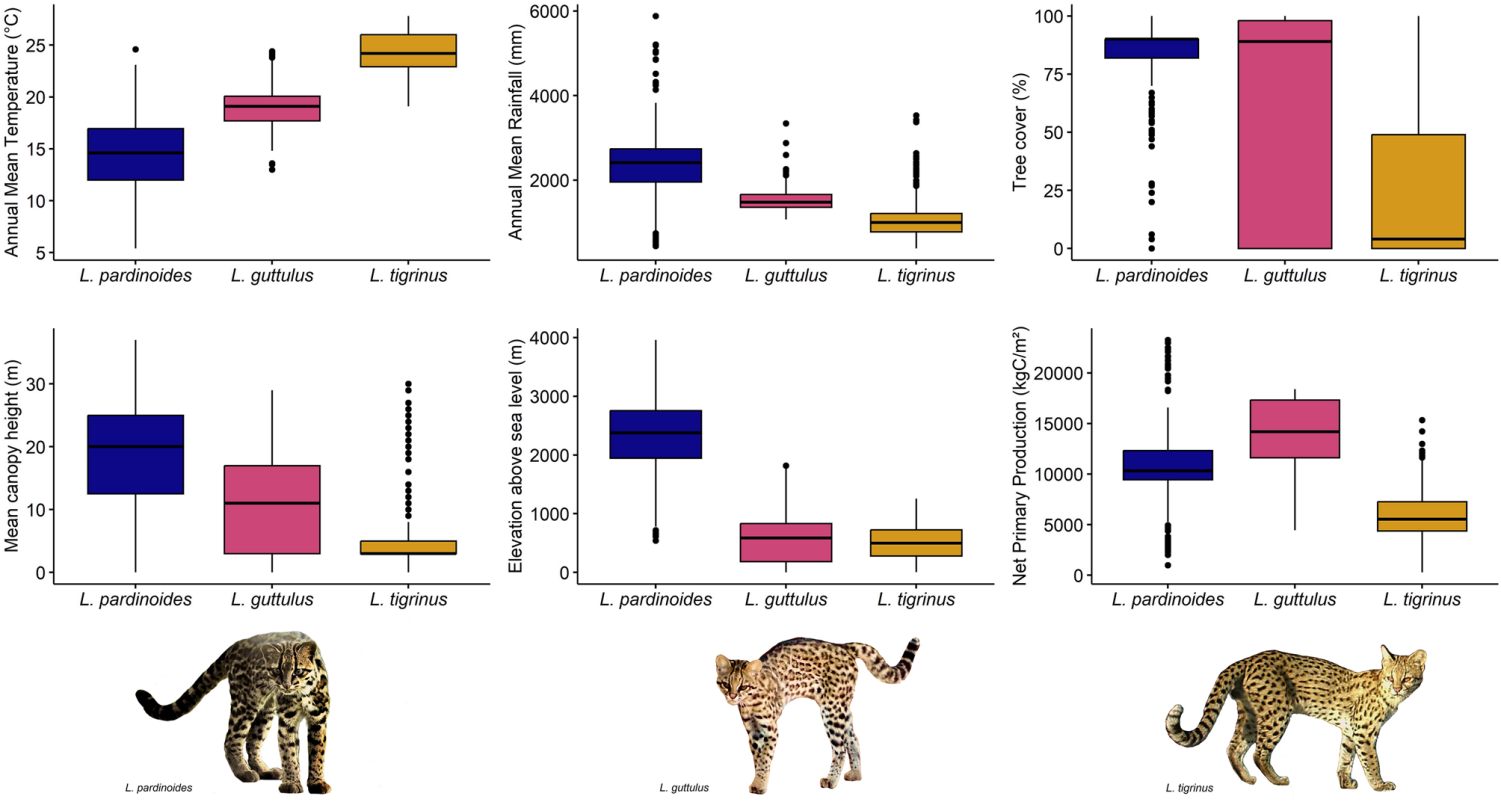
Multidimensional niche parameters showing the variables with higher impact that shape the hyperdimensional niche of the tiger-cats. Photographs: (**a**) authors, (**b**) Weslley Alves, (**c**) authors.

Of the 25 continuous variables assessed (Supplementary Table S4), we choose six to contextualize the multidimensional environmental universe of each species of the complex: canopy height, elevation, mean annual rainfall, mean annual temperature, net primary production, and tree cover (Fig. 7, Table 2). For most of the variables, there was little to no overlap among the species for the median, 25th percentile, or 75th percentile. In our models, the temperature and the altitudinal range were correlated, prevailing the former. However, both are key elements in understanding the species. The mean annual temperature is mild for clouded tiger cat (ca. 15 °C), warm for the Atlantic Forest tiger-cat (ca. 20 °C), and hot for the savanna tiger-cat (ca. 25 °C). The altitudinal range is a key element describing the multidimensional realm of the clouded tiger-cat. We have records going from 540 to 3960 m asl, for which the median lies at 2379 m and the bulk of the records between 2000 and 3000 m asl. The other two species are essentially lowland forms, with a median (range) altitude of 497 m (3–1254 m asl) for the savanna tiger-cat and 585 m (0–1817 m asl) for the Atlantic Forest tiger-cat. The annual rainfall shows that the savanna tiger-cat is found in dry weather environments, whereas the Atlantic Forest tiger-cat is found in wet environments, and the clouded tiger-cat in very wet environments. Climate-wise, the savanna tiger-cat is from the tropical savanna dry (AW) or semi-arid areas (BSh), whereas the Atlantic Forest tiger-cat is from sub-tropical sites with hot (Cwa, Cfa) or mild (Cwb, Cfb) summers, and the clouded tiger-cat is from areas with sub-tropical mild to temperate cold summer weather (Cwb, Cfb/Cfc, ET; S5 Table). An interesting pattern is that net primary production (NPP)/GPP, which are measures related to prey availability^35^, indicate that the Atlantic Forest tiger-cat is found in the most productive lands, followed closely by the clouded tiger-cat, with the savanna tiger-cat found in the least productive soil. The percentage of tree cover clearly shows that the clouded tiger-cat and Atlantic Forest tiger cat are forested species. Their median value is very similar (90% and 89% tree cover, respectively); however, their range (25th–75th percentile) is very different, with values ranging from approximately 80–92% for *L. pardinoides* but 0–100% for *L. guttulus*. The median and percentile range for the savanna tiger-cat shows that it is from non-forested formations (4%, 0–50%). Although both are forest species with overlapping canopy cover, the approximate canopy height varied from 20 m for the clouded tiger-cat, to 12 m for the Atlantic Forest tiger-cat, and only 3 m for the savanna tiger-cat.

#### Multidimensional diagnosis of the species

**Figure 8:**
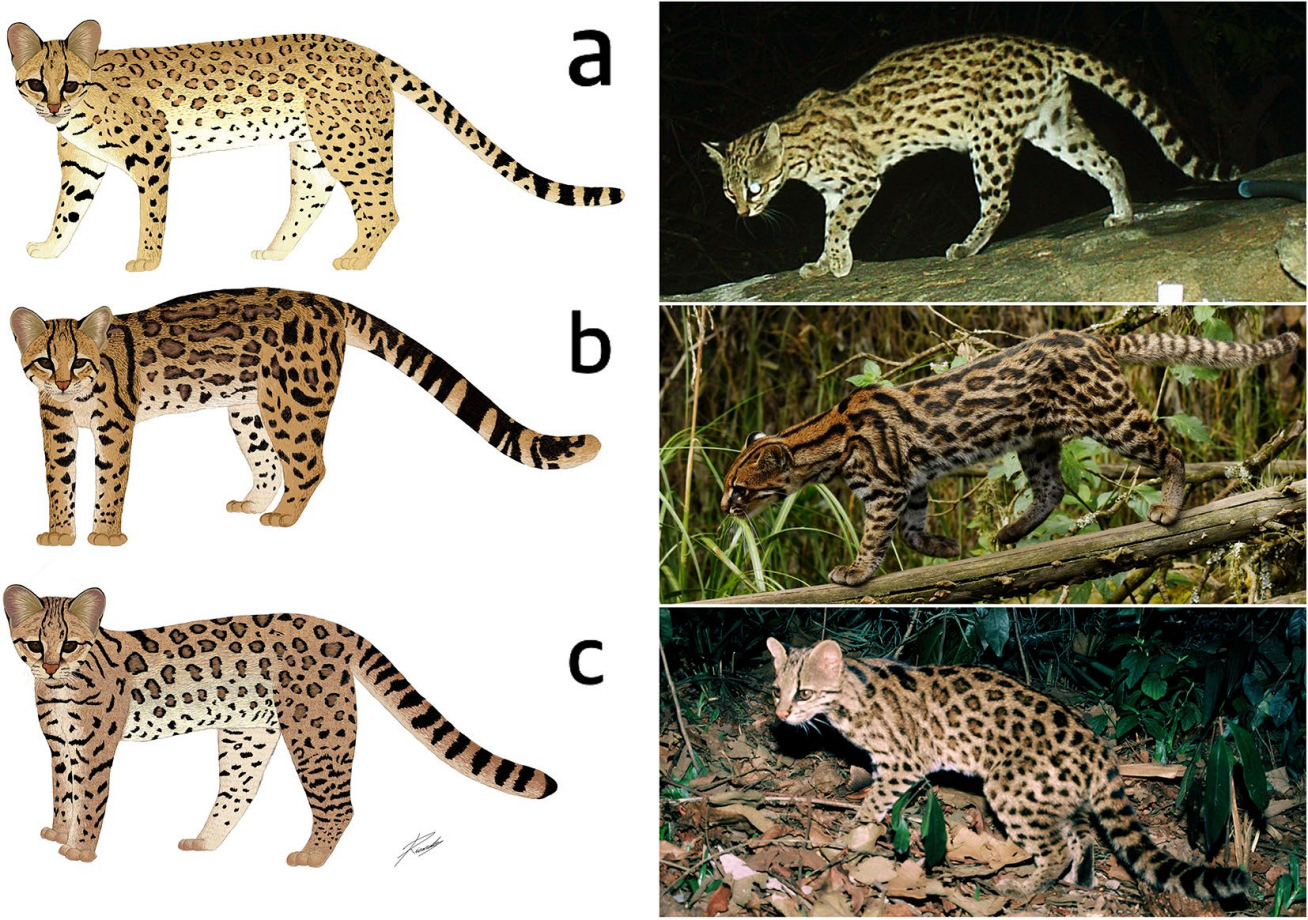
Classic examples of the tiger cat species complex: (**a**) the savanna tiger-cat (*Leopardus tigrinus*); (**b**) the new species, the clouded tiger-cat (*Leopardus pardinoides*); and (**c**) the Atlantic Forest tiger-cat (*Leopardus guttulus*). Drawings by Ricardo Ribeiro; photographs: (**a**) authors, (**b**) Johannes Pfleiderer, (**c**) authors.

*Leopardus tigrinus* (Schreber, 1775^34^): The savanna tiger-cat is a 2.34 kg felid with a slender long-legged body and long margay-sized thin tail, with proportionally large ears, and a yellowish or gray-yellowish background color with either small open or solid large dot-like rosettes that may coalesce, with two pairs of mammae/teats (Fig. 8a). The savanna tiger-cat range comprises varying vanishing savanna and dry scrub woodland formations in Brazil and the Guiana Shield, which means that they mostly live in lowland non-forested habitats with low tree cover (typically < 50%), with a median canopy height of 3 m that nevertheless has dense undergrowth, and that are mostly found in less productive soils, in areas of dry weather (< 1000 mm rainfall/year) with hot temperatures, and in tropical dry/semi-arid climates, typically where the dominant mesopredator/intraguild killer (ocelot) is absent or low in number. Intraguild interactions appear to play a role and to have evolutionarily limited the species multidimensional space.

*Leopardus pardinoides* (Gray, 1867^36^): The clouded tiger-cat is a long-tailed 2.27 kg cat with short-round ears and a remarkably margay-looking head, which has a nice dense soft fur of a rich reddish/orangish/grayish-yellow background color adorned with irregularly shaped medium-large “cloudy” rosettes that are strongly marked and often coalesce. Distinctively, the species has only one pair of mammae/teats (Fig. 8b). The clouded tiger-cat is found in the vanishing cloud forests of the southern Central American and Andean ranges at typically above 1500 m asl, but especially between 2000 and 3000 m asl, where tree cover is very high (90%), with a canopy height of 20 m, in fertile soils, in a sub-tropical/temperate climate with mild temperatures and very abundant rainfall, typically in areas where ocelot numbers are low or absent. The species may have been evolutionarily limited by intraguild interactions.

*Leopardus guttulus* (Hensel, 1872^37^): The Atlantic Forest tiger-cat is a 2.38 kg cat with a medium-large, bushy, thinly ringed tail, with a slightly bulky body adorned with large and numerous round rosettes in a background color with typically (but not restricted to) reddish/brownish-yellow tone, and has two pairs of teats (Fig. 8c). The species is found in the dwindling forest formations of the Atlantic Forest biome, with tree cover of 89% (although with a high variation, 0–100%) and a canopy height of 12 m, mostly in lowlands and areas of fertile soils in tropical/sub-tropical sites with either hot or mild summers with high rainfall and warm temperatures, notably where the ocelot is either absent or present in low density. Intraguild interactions appear to play a limiting role in the species multidimensional space.

## Discussion

### Biogeographic issues

The distribution assessment of the entire tiger-cat complex revealed a completely different arrangement than any of the distribution maps presented previously^4,5,10,12,13^. Recently, an analysis had already indicated *L. tigrinus* as absent from the rainforests of most of the Amazon biome, with the exception of the savanna’s enclaves, the Guiana Shield, and eastern Amazonia, east of the Tocantins river^9^. An analysis conducted for Colombia under the *L. tigrinus* name restricted the distribution of tiger-cats in the country to the three Andean cordilleras^38^.

Our model predicts high suitability for *L. pardinoides* in three mountain ranges of the Darién gap: Serranía de Jingurudo, Serranía de Pirre, and Serranía de Darién. The first two are geologically an extension of the northern Andes^39^. Although no records of the clouded tiger-cat has been obtained there, future surveys in the area targeting the species should be conducted to confirm its presence. In the past, there have been specimens of Andean bears (*Tremarctos ornatus*) collected in the Serranias, however current assessments recorded only claw marks, leaving doubts to its de facto presence there^40,41^. Thus, it could be possible that the clouded tiger-cat could be found in this rugged and unexplored area. Meanwhile, we predict that the eastern Panamanian montane forest specimens are more likely to belong to the South American *L.p. pardinoides* subspecies, whereas *L.p. oncilla* is likely to be restricted to the southern Central American montane cloud and elfin forests.

Our proposed historic maps intended to correct the prior inaccuracies surrounding tiger-cat distribution, given the lack of knowledge at the same time, by comparing to the most likely current possible range. They contrast sharply with the currently most acknowledged and used range maps of the IUCN^12,13^. The differences were all a result of range restrictions for all species of the complex. The major reductions in area size for the savanna tiger-cat arise partly because of the removal of almost all the Amazon basin, and partly as a consequence of the rampant habitat removal of the Caatinga and especially the savannas of the Cerrado biome. For the Atlantic Forest tiger-cat, the reduction is due to the losses of deforestation and for habitat restructuring, which removed the Cerrado biome from most of its range and limited it notably within the threatened and highly deforested Atlantic Forest domain. For the clouded tiger-cat, the area east of the Andes all the way to the Cerrado domain borders would be out, as this at the time was under *L. tigrinus*^3^.

As previously shown by de Oliveira et al.^42^, and confirmed again here, savanna tiger-cats are absent from the rainforest formations of Amazonia (except for the Tocantins forest ecoregion), and are restricted mostly to its savanna enclaves. The difference in the range in Amazonia between our model and that of de Oliveira et al.^42^ relates to the different approaches used (MAXENT vs. GLM models). Additionally, in our land cover variable we excluded open savanna formations, resulting in large areas of the Rupununi and Sipaliwini savannas unsuitable for savanna tiger-cats, whereas in de Oliveira et al.^9^, both areas in their entirety were classified as suitable. We could not properly model the ocelot effect as a variable, as performed by de Oliveira et al.^42^, because only GLM models account for the absence points necessary to properly reflect this effect.

The deforestation and process of savannization occurring in southeast Bahia and Minas Gerais states (Brazil) might provide some ground for closer contact between the savanna tiger-cat and the Atlantic Forest tiger-cat. To date, no hybrids of these have ever been detected^4^; even in captivity, they would not breed^43^. In the extreme north of the Atlantic Forest, where the remnant patches are degraded and there is a strong influence of the Caatinga, the species appears to have been replaced by *L. tigrinus*.

We also consider the proposed current map updated as only a small percentage of data was obtained from museum records (11.95%), i.e., most likely from the late 1800s and early 1900s. Without the use of other techniques, such as image identification from camera trapping (obviously only if associated with very accurate felid identification), this would not have been possible. Additionally, we had recent records (after the year 2000) from most of the areas close to those described in the museum records. An equivalent approach to ours, relying heavily on camera trapping records, was conducted for the short-eared dog, *Atelocynus microtis*^26^.

One potential limitation to our study is that presence-only approaches in species distribution models are sensitive to sampling bias^44^. The southern Andean region, particularly the Bolivian Yungas, as well as the Guiana Shield, are poorly represented in our samples. Access to these areas is logistically difficult, as they are far from permanent population centers or on very steep terrain. Additionally, the only long-term (≥ 15 years) camera trapping monitoring program that we are aware of in the Guiana Shield is in the rainforest site of Central Suriname Nature Reserve^45^. A suitable habitat for tiger-cats in the shield is restricted to savanna enclaves, where camera trapping studies have only recently been implemented^46,47^. With additional records from these areas, our models may have predicted a more suitable habitat there. At any rate, given the small area of the Guiana Shield in relation to the rest of the savanna tiger-cat range (Caatinga and Cerrado), increased suitability in the shield is not going to change the grim outlook for the species. Similarly, although the higher suitability of the Southern Andes would increase the potential range of the clouded tiger-cat, it would not result in a species with a lower threatened status as habitat loss is also an issue in the Southern Andes.

#### The Pantanal enigma

Our models showed limited suitable habitat for *L. tigrinus* in the Pantanal, along border areas with the Cerrado, and no suitable habitat for *L. guttulus*. There were some location points of the savanna tiger-cat right at its border area (Supplementary Fig. S2). An extensive camera trapping assessment conducted in several areas there did not record it^48^. Additionally, the only alleged camera trap record^49^, at closer inspection, was undoubtedly proven to be of an ocelot. However, the authors claimed to have observed animals that resemble Atlantic Forest tiger-cats. However, specimens of *L. tigrinus* found in the northern, central, and western savannas can exhibit a rosette pattern with some resemblance to *L. guttulus* (Supplementary Fig. S3). If the skin collected was indeed from an animal from the Pantanal and in line with the observations made by experienced wildlife specialists at forested areas, there is also the possibility that that there would be a relictual population of the Atlantic Forest tiger-cat there, similar to the Guiana Shield population for the savanna tiger-cat. Nevertheless, for the Atlantic Forest tiger-cat to be the de facto species found in the biome, the only possibility would be for it to have colonized the Pantanal in ancient times, likely during glacial or interglacial periods^50^, which is not in any way likely by today’s bioclimatic configuration. Meanwhile, if tiger-cats are indeed found there, there is a much higher probability that it will be the savanna tiger-cat that is found in the dry high-ground savannas. Interestingly, Tortato et al.^8^ postulated that, given the high number of ocelots in the Pantanal, the ocelot effect (the negative impact of the dominant mesopredator) could be the reason that there may be an extremely low number of tiger-cats there, to the point of being extremely hard to detect on camera, with perhaps sporadic incursions or being kept out of the biome altogether. In summary, if a tiger-cat is indeed in the Pantanal or only has sporadic incursions, and which species would be found will remain an enigma until hard evidence can be gathered. Additionally, the possibility of both a relictual *L. guttulus* population in forested areas and the incursions/presence of *L. tigrinus* in dry Cerrado savannas, both limited by ocelot abundance, is also possible, although unlikely.

#### The Guiana shield–Brazil connection

Traditionally, the taxonomy of the tiger cat has always considered the Guiana population and those of northeast Brazil under the same subspecies, i.e., *Leopardus tigrinus tigrinus*^1,2^. However, Nascimento and Feijó^7^ considered the NE-Brazilian population as a distinct species under *Leopardus emiliae*, placing the Guiana population along with those of northwestern South America and Central America under the species *L. tigrinus*. The other taxonomic scheme considers *L. tigrinus tigrinus* as a species of Guiana/Brazil, whereas *pardinoides* and *oncilla* are the subspecies of NW-South America and Central America, respectively^1–3^. Thus, we ask why is the Guiana pattern so pivotal in tiger-cat systematics? Because the species holotype is from Cayenne, French Guiana, which means that where this population is, it has the species name *tigrinus*.

We performed a PCA analysis of the Guiana specimens with those of the eastern Amazonia and the northern savannas and with those of *L. pardinoides*. The Guiana Shield specimens grouped with those of Brazil, and not with *pardinoides* (Fig. 2). This means that where ecological niche modeling is concerned, and skin patterns (see below), they belong to the same group and, as such, would invalidate the proposal of *Leopardus emiliae* as the species for northeastern Brazil^7^.

Skin patterns of the Guiana specimens are completely different from those of NW-South America (i.e., *pardinoides*) and those of Central America (i.e., *oncilla*), but very similar to those found in eastern Amazonia and northeast and central Brazil (Supplementary Fig. S4). Live specimens in both areas were also remarkably alike (Supplementary Figs. S5, S6). Additionally, the Guiana population might have been colonized from Central Brazilian specimens during the forest/savanna contraction/expansion during interglacial times over the Pleistocene^42^ (Supplementary Fig. S7). Bonilla-Sánchez^51^ also found some support for this and especially for the fact that there was no possible dispersion between the Andean population of *pardinoides* and that of E-Venezuela and of the Guiana Shield. To end this controversy, genetic confirmation is still required, but all the evidence provided leads to only two options: either the Guiana Shield and the Brazilian population are grouped together (as suggested by all evidence presented here) or the Guiana Shield comprises a distinct group by itself.

### The multidimensional space

Several approaches toward niche dimensions have focused on certain aspects of the niche—morphological, climatic, or simply depicting the hypervolume geometry^23^, but an integrative manner has not been used. Very few studies have used the multidimensional approach or something close to it^31,52^, but did not fully describe its multidimensionality.

There are hard-to-measure dimensions that certainly impact the niche space, such as interspecific relationships. This is another dimension that we believe impacts or has impacted tiger-cat occurrence that could not be measured. Intraguild relationships, through either potential killing or competitive exclusion, could have even evolutionarily prevented or limited the proper establishment of tiger-cat species. The ocelot effect, caused by high ocelot abundance in the Pantanal, might prevent the proper establishment of tiger-cat populations there^8^. Similarly, high ocelot densities in the Amazon rainforest were the main factor limiting the occurrence of *L. tigrinus* to the savanna enclaves in the biome^42^. High ocelot density in the Amazon may have been a possible ecological barrier in the evolutionary process, as *L. tigrinus* might have been eliminated in the areas where the ocelot was abundant; thus, the species might not have evolved adaptations to fully colonize the Basin, instead remaining only in its outskirts and where there was a low density of the mesopredator. We speculate that the ocelot effect might also limit the clouded tiger-cat below ca. 1500 m asl. At 2000–3000 m asl, low ocelot numbers had no effect on the species^20^, but this was different at lower altitudes when ocelot abundance increased^53^. The Atlantic Forest tiger-cat is not restricted geographically by ocelots. However, the ocelot densities are smaller in the Atlantic Forest domain than in the Amazon^42,54^. In any event, at sites of high ocelot abundance, tiger-cats and other felids are either absent or low in number^54–56^. Geoffroy’s cat (3.7 kg in S-Brazil), through competitive exclusion, and perhaps even the *geoffroyi/guttulus* hybrids appear to be the limiting factor for the southern limits of the Atlantic Forest tiger-cat being at the Central Depression of Rio Grande do Sul and not reaching the theoretically suitable forest areas of the Pampa biome that would extend all the way to the Uruguayan border^57^. Geoffroy’s cat could also have historically limited the expansion of the savanna tiger-cat into the Chaco, which has similar environmental conditions and shares close biogeographic affinities to the other environments of the dry diagonal, Cerrado and Caatinga^58–60^, where *L. tigrinus* is found.

The NPP/GPP dimension and its relationship with prey availability^35^ affect species abundance. Thus, our results showing the Atlantic Forest tiger-cat in the most productive lands, followed closely by the clouded tiger-cat, and the savanna tiger-cat in the least fertile land, should be reflected in their overall abundances (although there are other factors also affecting this). The abundance indices of these species followed the patterns of their mean NPP values: 1.13 ind./100 trap-nights (*L. guttulus*, *n* = 12), 0.86 ind./100 trap-nights (*L. pardinoides, n* = 7), and 0.58 ind./100 trap-nights (*L. tigrinus, n* = 11)^61^.

We compared the canine diameter in all tiger cat species as a metric to indicate if they would be preying in different class size of prey, and our results suggested that they are not (see Supplementary Information). They should all be preying on the smaller spectrum of prey, which consists of varying degrees of small rodents and marsupials, small birds, and lizards^48,55^. To complete their ecological niche dimension, all species had predominantly nocturnal activity, with varying degrees of diurnal activity that were often associated with the avoidance of the dominant ocelot^55,62–66^.

### Genetic support

The available genetic information on *Leopardus* and especially on the tiger-cats reflects a complex history. Lescroart et al.^17^ used a molecular genomic analysis to show that specimens from the Colombian Andes (*L.t. pardinoides *sensu Wozencraft, Kitchener et al.^2,3^) were related to those from Costa Rica (*L.t. oncilla *sensu Wozencraft, Kitchener et al.^2,3^). The differences detected were still shallow for species differentiation belonging to the same group. The name *pardinoides*^36^ has taxonomic prevalence over *oncilla*^67^; henceforth, they should be grouped under *pardinoides* as the species/group name. However, there was discordance between the genomic phylogeny and mitogenomic maximum likelihood phylogeny with bootstrap support. Either way, *pardinoides/oncilla* remains as one group. Both schemes indicate that given the current molecular knowledge, tiger-cats (sensu Cabrera, Kitchener et al.^1,3^) are not a monophyletic group^16,17,68^. The split between *L. tigrinus* and *L. guttulus* happened 1.52 MYA (1.05–1.9), whereas their ancestral group, which included *tigrinus/guttulus* and *geoffroyi/guigna* groups, split from *pardinoides/oncilla* 2.45 MYA (1.73–2.97)^16,17,69^. Another very interesting yet worrisome point relates to mean autosomal heterozygosity, which was 0.16% for *guttulus*, 0.13% for *pardinoides,* 0.08% for the *oncilla* subspecies, and only 0.05% for *tigrinus*. The values for *guttulus* and *pardinoides* (Colombia) are similar to those for the margay and Geoffroy’s cat (ca. 0.14%/0.15%), but smaller than for the ocelot (0.27%). Notwithstanding, that of the restricted Central American tiger-cat population (*L. pardinoides oncilla,* < 26,000 km^2^) was almost twice as much as that of the wide ranging *L. tigrinus* (ca. 1.5 million km^2^), which, in turn, was larger only than that of the Andean cat (*L. jacobita*), for which the low level (0.02%) is indicative of a long-term small population size^16,17,69^. This low level for the savanna tiger-cat should be of concern regarding conservation, and could have been a result from the bottleneck likely at the time of the introgression with the female Brazilian Pampas cat (*L. braccatus*^4^).

In summary, the molecular support to place *pardinoides/oncilla* as an altogether different group of tiger-cats from *L. tigrinus* and *L. guttulus* was confirmed by Lescroart et al.^17^. Interestingly, when Thomas^67^ originally described *oncilla* he placed it as a subspecies of *Felis pardinoides*, rather than as a species on its own, which has eventually been proven correct, as confirmed by the recent genetic findings and our niche modeling.

### Conservation implications

The reduction in area from the historic range to the current range for all three species is a massive − 3,173,402 km^2^, an area slightly smaller than India or representing approximately 75% of the European Union. It is highly important to mention that these red alert area reductions are for species that, where found, typically show low or very low abundance^12,13,19,20,70,71^. Given the historic loss of the Atlantic Forest domain^72^, current area reductions would likely be greater for the savanna and clouded tiger-cats. Their serious area reductions would place their separate global red list assessments by IUCN at a high priority level. In this scenario, three countries emerge as key for the conservation of the species complex. Brazil holds 93% of the current range of the Atlantic Forest tiger-cat and 98% of the savanna tiger-cat; Colombia is key for the clouded tiger-cat with approximately 40% of the species range and holds key areas for their conservation in all three cordilleras^20,38^; Costa Rica is a key area for the Central American subspecies of clouded tiger cat (i.e., *L.p. oncilla*) with more than 60% of its range. Given the current rates of habitat loss in the Andean cloud forests and in the “new” agriculture frontier of Brazil, the MATOPIBA savannas (the northern savannas) and the Caatinga of Brazil, the core of the savanna tiger-cat range, *L. tigrinus* and *L. pardinoides* would be the species under more intense pressure. It has been suggested that the path that led the Pampa cat (*Leopardus munoai*) to the brink of extinction, given the high rates of native habitat conversion to large-scale agriculture (grains), is already being followed by the savanna tiger-cat^73^. Additionally, the occupancy of all three species is negatively affected by proximity to human settlements/households^18,20,74^, which creates another problem given the fragmentation process and the ever-increasing human encroachment.

All these factors lead to a dire need for real conservation policy action, notably—but not, by any means, exclusively—for Brazil and Colombia. Thus, the fate of both savanna and Atlantic Forest tiger-cats lies in the hands of Brazilian authorities. In theory, this is less complicated, as no agreement between countries is required. For the clouded tiger-cat, conservation will be much more complex, as it also involves conflict (guerrillas) areas. For Brazil, public policy would perhaps require a change in the law for mandatory non-forested property’s designated protection area from 35 to 50%. Realistically, this is almost impossible as the land is the new agricultural frontier and it would be prevented by the agribusiness lobby. This means that methods of conserving small cats in private properties will become crucial. Having a method of integrating the 35% already required by law by different landowners would be a huge task for CAR (Brazil’s regulatory “Rural Environmental Registry”), but is very much needed, as it would maximize better connectivity among the remaining areas in both forested and non-forested environments. One final and important issue for the savanna tiger-cat population relates to its already very low levels of heterozygosity^69^ that, in association with the high levels of habitat conversion and fragmentation/isolation^75^, result in a very worrisome outlook for this species.

## Concluding remarks

Only after the advent and improvement of camera trapping were we able to start noticing the live specimens of clouded tiger-cats (*pardinoides* and *oncilla*) and realize that they could be distinct species. This provided a completely different perspective than that of museum specimens. Without the “flesh”, live contour, and proportions, the real appearance of the live animal cannot be observed; thus this is not possible from a museum pelt or skull. We believe that this might have prevented it from being considered a distinct species, until now. Indeed, we believe that the clouded tiger-cat is the species most distinct from the others in appearance, which can now be explained by its larger phylogenetic split from the other two species^16,17^.

Camera trapping photographs and video has provided a very insightful means of depicting species variability as a powerful complementary option for museum collections without the need to sacrifice or remove any animal from nature. In today’s digital and technological culture, it would prove very useful to have photographic records of all museum specimens available digitally (through institutional catalogs) alongside camera trapping and other photographic/video records. However, correct identification from the photographic material by very experienced specialists is crucial. This is a very limiting issue for Neotropical small cats. The misidentification of *Leopardus* species is very common^42,76,77^. Photographs, morphological, and skin pattern portrayals of the complex in books and guides usually mix them up and do not contain accurate or proper depictions. However, our approach of using personal photo-records of museum specimens and thousands of camera trap images of the small Neotropical felids, backed by previously obtained sound knowledge of the animals in museums, zoos, and nature were pivotal for identifying cryptic-looking species of the complex *Leopardus* genus. Ultimately, all the records used in this analysis are guaranteed to have been properly checked and verified; we did not use questionable records.

We have provided a robust body of evidence based on the morphology, phenotype, biogeography, and niche modeling to properly set and characterize *Leopardus pardinoides*^36^ as a valid species apart from *Leopardus tigrinus*^34^ and *Leopardus guttulus*^37^, backed by strong genetic support^16,17^. We also innovated by taking the phenotypic/taxonomic approach to use photographic material as means to aid the establishment of species patterns beyond traditional and exclusively museum specimens. This is the first time that a species multidimensional space is plainly described in morphologic, phenotypic, environmental, and ecological terms together, i.e., the real high-dimensional biological hypervolume that not only defines a species, but also was used to elucidate a multi-species complex, elevating and validating a new felid species, while differentiating their n-dimensional hypervolumes from one another. The biogeographic assessment conducted presented a completely new arrangement of the historic and current distribution of the threatened tiger-cat species complex, finding that the arrangement was very biogeographically oriented for all species, with the new clouded tiger-cat (*L. pardinoides*) from the cloud forests of southern Central America and Andean mountain ranges, the savanna tiger-cat (*L. tigrinus*) from the Guiana Shield and the savanna and dry shrub-woodland formations of central-northern Brazil, and the Atlantic Forest tiger-cat (*L. guttulus*) from the Atlantic Forest domain. Given their ranges, Brazil, Colombia, and Costa Rica should take key roles in conservation of the species complex. For all tiger-cats, very concerning area reductions, ranging from − 50.4 to − 68.2%, were identified. This also requires immediate action, with enhancement and reinforcement of environmental policies, an urgent need for Red List assessment/reassessment for all species, in addition to taking strong conservation action from local projects to national institutions and authorities. The “Red Alert” is on.

## Materials and methods

### Tiger cat species complex occurrence points

We gathered all records we could find of both species using the following sources: camera trap photos from studies across species’ respective ranges (published and unpublished); museum records with georeferenced locations; roadkill; and verified visual observations. Each record was assessed for its veracity, and only verified records were included in the analysis. Records without verified coordinates were also excluded from the analysis (i.e., a few apprehended specimens with no known origin). In total, we assembled 1439 presence records at unique locations, of which 1105 were used for the subsequent analyses of distribution (see “Modeling procedures” section).

### Species identification and their phenotypic patterns

To establish the patterns of species variation, we combined personal photographic records of museum specimens, notably from the Zoology Museum of São Paulo University (MZUSP), the American Museum of Natural History, the National Museum of the Smithsonian Institution (NM); we were also able to check some online photo-records of the Leiden Museum and a few others from the literature, especially from some small local collections in Costa Rica, Colombia, and Brazil. Specimens from several other museums/collections were also checked, although without a “photo reminder”. The amount of pictures for species identification received also contributed considerably. However, the bulk of the material used came from camera trapping studies. Record points used here often times would represent several specimens, so that the amount of photographs seen would escalate enormously. For example, over 600 pictures, representing ca. 80 independent records (i.e., records > 1 h apart) of clouded tiger-cats from Ecuador, sometimes with 5–7 sets of pictures showing the same individual at several positions helped to build a mental algorithm on how they looked like. This was crucial for getting to know and building up the looks of the enigmatic clouded tiger cat, previously known from flat museum skins and not from living animals. In the end, the lead author estimated having seen easily > 2000–3000 “records” of tiger-cat complex specimens from pictures, videos, museum skins, skulls, and of observing living animals of *tigrinus, pardinoides, oncilla*, and *guttulus*. If other species of *Leopardus* are included, numbers would soar way above 6000. All this provided the experience to perform accurate species identification of all records. We only used records properly identified/checked by us (TGO), disregarding any doubtful records.

Morphological data were obtained from personal and institutional archives. No animal was trapped or handled for the purpose of this work, we are simply using existing data from several authors.

We also examined how the Guiana Shield specimens would compare to both *tigrinus* and *pardinoides*, to test the validity of *L. emiliae* (sensu Nascimento and Feijó^7^).

### Explanatory variables

To model the potential distribution of each subspecies of the *L. tigrinus* complex, we extracted 27 bioclimatic, environmental, and topographic variables: 19 bioclimatic variables from the Worldclim dataset ver. 2^78^, elevation and terrain ruggedness from Shuttle Radar Topography Mission^79^, net primary production (NPP) and gross primary production (GPP), derived from MODIS^80^, percentage tree cover^81^, canopy height^82^, land cover^83^, and ecoregions^84^. For detailed information on each variable (description, source, and year), see S6 Table. The land cover layer includes 11 land cover classes, which we reclassified into the following: (i) tree cover, (ii) shrublands, (iii) unsuitable habitat (for further details, see Supplementary Table S7). We included the ecoregion variable to account for unmeasured differences in vegetation, water availability, and other environmental features among the different ecoregions.

All variables were standardized to a 30 arc-s resolution (approximately 1 km at the Equator). We chose this resolution to account for the fine-scale habitat selection and climate variability. At coarser resolutions, small areas of suitable habitat disappear, leading to biased predictions of distribution^85^. This would be especially problematic in areas such as the Atlantic Forest, where the landscape is highly fragmented. Prior to modeling procedures, we calculated Pearson correlations among all numerical predictors. For the pairs of predictors in which r ≥|0.70|, we included the one with the highest contribution to the PCA (see “Modeling procedures” section).

### Morphological traits

We assessed the following morphological traits for each subspecies: body mass, head and body length (HB), tail length (TL), TL/HB proportion, ear size (E), E/HB proportion, and upper canine (antero-posterior) diameter. We collected these measurements from a total of 104 specimens (*L. t. pardinoides/oncilla* = 21; *L. t. tigrinus* = 22; *L. t. guttulus* = 61). Not all measurements were available for all specimens (see below). Additionally, we performed a descriptive comparison of body pattern, color, and spot patterns.

### Statistical analysis

#### Modeling procedures

Before constructing models, we conducted a principal component analysis using all records in the dataset and all numerical predictors. We standardized all predictors to a standard deviation of one and a mean of zero, in order to make them comparable as the units of measurement differed between some predictors. We grouped records based on the subspecies as follows: *L. tigrinus tigrinus, L. tigrinus pardinoides, L. tigrinus oncilla,* and *L. guttulus*. Because the PCA showed a complete clustering of *L. t. oncilla* records within *L. t. pardinoides*, we grouped both subspecies as *L. t. pardinoides* for subsequent analyses.

We modeled the habitat suitability of each group using the maximum entropy algorithm in Maxent 3.4.4^86^. This algorithm uses presence-only data as well as background points obtained from a geographic area defined a priori. Using the species locations and the covariate values, the algorithm estimates the target probability distribution from the principle of maximum entropy (i.e., finding the distribution closest to uniform). Maxent has been widely applied to model species distribution and habitat suitability, thereby identifying priority areas for conservation as well as delineating species ranges^26,29^. Although other presence-only algorithms exist for modeling species distributions, Maxent has consistently been shown to make robust predictions and outperform other algorithms^87–89^.

For each group, we conducted a PCA to identify which variables maximized variability among the records’ locations. For each PCA, we selected the most important variables. For variables that were highly correlated (r ≥ |0.70|), we retained the variable that contributed the most to a specific PCA. The categorical variables (ecoregion and land cover) were included in the models for the three groups. To define the geographical area for projecting the models, we considered both the records and nearby ecoregions. For all three groups, we extended the geographical space to include areas where occurrence is uncertain, as we believe this could help pinpoint the range limits of each species. Specifically, for *L. t. tigrinus*, we included the entire Amazon basin, plus the Cerrado and Caatinga biomes, as well as transitional areas with the Atlantic Forest. For *L. guttulus*, we considered all tropical and sub-tropical moist broadleaf forest ecoregions in southern Brazil, eastern Paraguay, and northeast Argentina; we also included the Uruguayan savannas and a 100 km inset in the Cerrado from the northernmost and westernmost records. Finally, for *L. t. pardinoides*, we projected models to all Andean forest and nearby Amazonian ecoregions, reaching the northern limit at the Nicaraguan depression. We applied a 3 km spatial filter to all records in our dataset to avoid spatial dependence among points and biases toward areas with greater sampling effort, resulting in 493 records for *L. t. tigrinus*, 208 for *L. t. pardinoides*, and 404 for *L. guttulus*.

For each group, we used 70% of the data for training the model and 30% for testing. We used 500 iterations, 10,000 background points, and a regularization multiplier of 1. Data subsets were generated using bootstrapping with 10 random partitions with replacements. We used a prevalence of 50%^90^.

To define the distribution of each subspecies, we reclassified each suitability map according to a threshold. Maxent provides several model thresholds, and we considered the following three thresholds when seeking to minimize test omission: maximum test sensitivity plus specificity; minimum training presence; and tenth percentile training presence. For all models, we obtained the area under the receiver operating characteristic curve (AUC), with models being considered acceptable if AUC > 0.70. We also measured the test omission rate, considering acceptable omission rates to be < 0.15, and the binomial probability, which we considered acceptable when *P* < 0.05. These measures were obtained for the average of the ten model runs.

Species distribution models will sometimes generate patches of suitable habitat in areas where the species is absent or prevented from colonizing by natural barriers^86^. Thus, we excluded from the maps all “suitable areas” that were located in areas beyond the known historic distribution of each species (i.e., small forest areas in Uruguay for *L. guttulus* and savanna-like areas in the Atlantic Forest for *L. tigrinus*). To determine the historic range we considered the full extension of the data points in a biogeographic approach using the ecoregion limits, as modelling results showed it to play a pivotal role in determining the species range.

To further assess differences in environmental presences between the subspecies, we conducted a multivariate analysis of variance (MANOVA) using 25 numeric bioclimatic and environmental variables. We then conducted a one-way analysis of variance (ANOVA) for each specific variable. We standardized all variables prior to analyses and used a significance level of 0.05. All analyses were conducted in R ver. 4.2.3^91^.

#### Morphological measurements

We first tested for the normality of all measurements using the Shapiro–Wilk test with a significance level of 0.05. For measurements that were normally distributed, we assessed possible differences between subspecies through one-way analysis of variance (ANOVA), otherwise, we used Kruskal–Wallis. On both cases, we used a significance level of 0.05. For the paired comparisons of species’ morphological traits, we performed a *t*-test, but for those in which we detected differences, we proceeded to perform a paired Mann–Whitney U test, again with a significance level of 0.05. All analyses were conducted in R ver. 4.2.3^91^.

### Supplementary Information

Supplementary Information.

## Data Availability

The datasets used and/or analysed during the current study are available from the corresponding author on reasonable request. A portion of the data (georeferenced data derived from museum records) will be provided by the first author pending scientific revision. The remaining of the data is not freely available because of conservation concerns. The species assessed in this study are globally threatened, hence exact locations are sensitive information. Data will be available, upon reasonable request to the first author, to qualified individuals who agree in writing not to publish or share exact locations or other information that could lead to harm or threaten the animals or areas where they live. All relevant information for replicating the statistics of morphological analyses is within the Supplementary Materials.
