# Supplementary material for: Ecological modeling, biogeography, and phenotypic analyses setting the tiger cats’ hyperdimensional niches reveal a new species
Source: Sci Rep. 2024 Jan 29;14:2395. doi: 10.1038/s41598-024-52379-8 (PMC10825201; doi:10.1038/s41598-024-52379-8)
Supplement: Supplementary file 1 — Supplementary Information. [file 41598_2024_52379_MOESM1_ESM.pdf]

Supplementary Material for

Ecological modeling, biogeography, and phenotypic analyses setting the tiger cats' hyperdimensional niches reveal a new species

Authors

Tadeu G. de Oliveira[1,2,3,4]*, Lester A. Fox-Rosales[3,5], José D. Ramírez-Fernández[3,6], Juan C. Cepeda-Duque[3], Rebecca Zug[7], Catalina Sanchez-Lalinde[3,8], Marcelo J.R. Oliveira[4,9], Paulo H.D. Marinho[3,10], Alejandra Bonilla-Sánchez[3,11], Mara C. Marques[3,12], Katia Cassaro[13], Ricardo Moreno[14], Damián Rumiz[15], Felipe B. Peters[2,3,16], Josué Ortega[14,17], Gitana Cavalcanti[18], Michael S. Mooring[19,20], Steven R. Blankenship[19], Esteban Brenes-Mora[21], Douglas Dias[3,4,22], Fábio D. Mazim[2,3,23], Eduardo Eizirik[2,11], Jaime L. Diehl[3], Rosane V. Marques[3], Ana Carolina C. Ribeiro[24], Reginaldo A. Cruz[25], Emanuelle Pasa[25], Lyse P.C. Meira[3,26], Alex Pereira[3,26], Guilherme B. Ferreira[9], Fernando F. de Pinho[9], Liana M.M. Sena[4], Vinícius R. de Morais[3], Micheli Ribeiro[3,27], Vitor E.C. Moura[3,28], Marina O. Favarini[2,3,16], Karla P.G. Leal[29], Paulo G.C. Wagner[30], Maurício C. dos Santos[3], James Sanderson[21,31], Elienê P. Araújo[32], Flávio H.G. Rodrigues[33]

Affiliations

[1]Universidade Estadual do Maranhão (UEMA), Dept. Biologia, São Luís, MA, Brazil.

[2]Instituto Pro-Carnívoros, Atibaia, SP, Brazil.

[3]Tiger Cats Conservation Initiative (TCCI), Brazil/Colombia/Costa Rica.

[4]Programa de Pós-Graduação em Ecologia, Conservação e Manejo da Vida Silvestre –ECMVS, Universidade Federal de Minas Gerais (UFMG), Belo Horizonte, MG, Brazil.

[5]UEMA, Programa de Pós-Graduação em Ciência Animal, São Luís, MA, Brazil.

[6]Oncilla Conservation, Costa Rica Wildlife Foundation, San José, Costa Rica.

[7]Universidad San Francisco de Quito, Quito, Ecuador.

[8]Onca Fundación para el Estudio de la Diversidad, Bogota, Colombia.

[9]Instituto Biotrópicos, MG, Brazil.

[10]Universidade Federal do Rio Grande do Norte (UFRN), Natal, RN, Brazil.

[11]Pontifícia Universidade Católica do Rio Grande do Sul (PUCRS), Porto Alegre, RS, Brazil.
[12]Zoológico de São Paulo Zoo, São Paulo, SP, Brazil
[13]Zoológico Beto Carrero World, Penha, SC, Brazil.
[14]Fundación Yaguará Panamá, Ciudad del Saber/Panama City, Panama.
[15]Noel Kempff Mercado Natural History Museum, Santa Cruz de la Sierra, Bolivia.
[16]Programa de Pós-Graduação em Biologia Animal, Universidade Federal do Rio Grande do Sul (UFRGS), Porto Alegre, RS, Brazil.
[17]Smithsonian Tropical Research Institute, Balboa Ancon, Panama.
[18]GAE Serviços Ambientais, Canto do Buriti, PI, Brazil.
[19]Point Loma Nazarene University, San Diego, California, USA.
[20]Quetzal Education & Research Center (QERC), San Gerardo de Dota, Costa Rica.
[21]Re:wild, Austin, Texas, USA.
[22]SETEG- Soluções Geológicas e Ambientais, Fortaleza, CE, Brazil.
[23]Ka'aguy Consultoria Ambiental, Pelotas, RS, Brazil.
[24]Universidade Federal de Goiás (UFG), Instituto de Ciências Biológicas, Goiânia, GO, Brazil.
[25]Cruzeiro do Sul Consultoria Ambiental Ltda., Ivoti, RS, Brazil.
[26]Bioconsultoria Ambiental Ltda., Caetité, BA, Brazil.
[27]Instituto Felinos do Aguaí, Siderópolis, SC, Brazil.
[28]UEMA, Programa de Pós-Graduação em Ecologia e Conservação da Biodiversidade, São Luís, MA, Brazil.
[29]Universidade Federal de Lavras (UFLA), Lavras, MG, Brazil.
[30]Centro de Triagem de Animais Silvestres CETAS, IBAMA-RS, Porto Alegre, RS, Brazil.
[31]Small Wild Cat Conservation Foundation, Corrales, NM, USA.
[32]UEMA, Núcleo GeoAmbiental, São Luís, MA, Brazil.
[33]UFMG, Dept. Genética, Ecologia e Evolução, Belo Horizonte, MG, Brazil.

* Corresponding Author:
Email: tadeu4@yahoo.com (TGO)

Characterizing the Species

Regardless their differences, all tiger cats show the distinctive signature of a lateral convex head profile and nape hairs not reversed, i.e., slanting backward from head to tail base (Supplementary Fig. S8). This sets them all apart from ocelots and margays, the other sympatric species of spotted *Leopardus*. For their cranial "signature," see section "Diagnostic differences from other sympatric *Leopardus*" here.

Leopardus pardinoides (Gray, 1867 [1])—Clouded tiger-cat: This small-sized felid has HB length of 484.86 ± 30.98 mm (*n* = 21), tail length of 290.43 ± 28.86 mm (*n* = 21), and body mass of 2.27 ± 0.46 kg (*n* = 10). It presents several external characteristics that are a mix of Atlantic Forest tiger-cat and margay, with the least resemblance to the savanna tiger-cat, the species it was supposed to be part of (i.e., *L. tigrinus sensu* Kitchener et al. [2]). The tail is bushy, long, margay-sized, and thus proportionally larger compared to the HB in ca. 70% of specimens. The head is very margay-looking (and easily confused by the eyes of an inexperienced observer), but despite the hard resemblance, the hair direction and lateral profile pattern are distinctive (if observable). The eyes look proportionally bigger than on the other tiger-cats, but still not as big and bulging as those of the margay. The ears are proportionally smaller compared to HB. The fore paws sometimes look proportionally large to the animal size, similar to the margay. The skin is not coarse, as described for the other tiger-cat species, but silkier, soft, with denser fur to the touch, likely to cope with the colder temperatures. Another margay-like feature is that females have only one pair of mammae/teats, and not two as in the savanna and Atlantic Forest tiger-cats, a very unique, interesting, and intriguing fact. The clouded tiger-cat does have highly skilled arboreal abilities, as captives can walk upside-down for short distances in enclosures, as margays do. This feature has not been reported in other tiger-cats. However, clouded tiger-cats do not have the ability to rotate the ankle joint, which is exhibited by margays.

The clouded tiger-cat has unique spot patterns, with irregularly shaped medium-large "cloudy/nebulous" rosettes that are sharply marked, and sometimes can coalesce in oblique lateral bands, as found in ocelots (Supplementary Fig. S9a). In contrast, some specimens (especially those in the southern part of the range) have rounder-like rosettes similar to Atlantic Forest tiger-cats, whereas very few have the rosettes all/almost all black, as also found in some margays and savanna tiger-cat (a pattern noted so far only in some specimens of the Peruvian

Yunga) (Supplementary Fig. S9b). Tail banding tends to be with few big black blotches, such as those of the margay and savanna tiger-cat, rarely with the abundant thin-rings commonly found in Atlantic Forest tiger-cat. The background color of the upper-parts tends to be rich fulvous, reddish-yellow/orangish-yellow or grayish-yellow, often with sharply defined black markings. The venter is white or with very light grayish-white tone [3]. Melanism is common, with at least 4.3% of our records of melanistic specimens. However, when we look at specific areas, it is a different scenario, with melanistic individuals representing 6.5%–32% of the records (Supplementary Fig. S10).

Leopardus guttulus (Hensel, 1872 [4])—Atlantic Forest tiger-cat or southern tiger-cat: This small-sized felid has an HB length of 509.61 ± 33.91 mm (n = 44), a tail length of 271.80 ± 21.44 mm (n = 45), and a body mass of 2.34 ± 0.45 kg (n = 46); in fact, it has the largest HB and the smallest tail of all tiger-cats. It tends to show a slightly bulkier appearance when compared with the other species. The ears are proportionally smaller to HB and rounder, such as the clouded tiger-cat, and not large as that of savanna tiger-cat. The tail is bushy, mostly medium-large (i.e., <56% of HB) in approximately 70%, with only approximately 30% having a proportionally large, margay-sized tail, the inverse of what is found in the clouded tiger-cat and the savanna tiger-cat. Females of this species have two pairs of mammae/teats.

Atlantic Forest tiger-cats have a greater external resemblance to the clouded tiger-cat, especially owing to their background color. Rosettes tend to be larger and rounder, especially when compared with those of savanna tiger-cat (Supplementary Fig. S11). However, there is a great deal of variation. In some populations in the state of Santa Catarina (Brazil) and on the southernmost limit near the hybridizing zone with Geoffroy's cat, rosettes can be smaller, more closely resembling those of the savanna tiger-cat. The ocelot banding pattern, where rosettes tend to coalesce to a higher or smaller degree, is present, but rarely found. Entirely black-dot rosettes have not been recorded do date. The typical medium-large bushy tail frequently shows numerous thin-rings, which tend to be distinctive from the patterns of both clouded and savanna tiger-cats. However, the blotchy patterns typical of the latter two are also found. Background color also varies, from rich tawny fulvous/reddish-yellow to ochraceous/brownish-yellow to a more grayish-yellow or yellowish-gray. This background color and spot pattern is distinctive from the savanna tiger-cat. The venter is white or with very light grayish-white tone. Although melanism

is not unusual, it is not common as in the clouded tiger-cat. Only 1% of our records were of melanistic individuals. Melanism apparently appears very opportunistically in Atlantic Forest tiger-cat, as suggested by multi-year/longer-term monitoring sites (0%–8.11%) (Supplementary Fig. S12).

Leopardus tigrinus (Schreber, 1775 [5])—savanna tiger-cat: This type species of the complex is a small-sized felid with HB length of 481.52 ± 56.32 mm (*n* = 21), tail length of 278.05 ± 31.71 mm (*n* = 20), and body mass of 2.32 ± 0.4 kg (*n* = 22). It tends to show two body patterns, one more of standard domestic cat shape with slightly longer limbs and medium–long tail (similar to *L. guttulus*) and often of a long-legged, slender body shape, appearing higher from the ground than the other tiger-cat species, with a long, thin tail. This often results in a large appearance for a small-sized cat. The tail is long, margay-sized, and thus proportionally larger compared to HB in ca. 65% of specimens. It differentiates from that of the Atlantic Forest and clouded tiger-cats as it is not bushy, but rather thin-looking; this is a distinctive feature. The tail is also proportionally larger than that of Atlantic Forest tiger-cat, but not of the clouded tiger-cat. The head has proportionally larger ears (42.56 ± 4.64 mm, *n* = 18), usually 1 cm larger than for the other tiger-cats, another distinctive characteristic. The rosettes have varying patterns (Supplementary Fig. S13); small and numerous open rosettes are standard, these can sometimes be shaped similarly tending to coalescing without actually merging, or coalescing on longitudinal oblique bands, similar to the ocelot banding pattern, which is not too common (Supplementary Fig. S14). A pattern that is commonly found in eastern Venezuela, the Guianas, and eastern Amazonia, but that also shows up in central and NE-Brazil, is that of rosettes forming slightly elongated spots, small or large, entirely black or not, which also could tend (or not) to coalesce to a smaller or larger degree (see Supplementary Figs. S3, S4, S5). Some specimens in the Cerrado savannas of northern, central, and western Brazil can have rounder and larger rosettes, similar to those of *L. guttulus* (see Fig. S2). The typical background color tends to be pale yellow, but darker tawny-yellowish colors areas are also found, especially (but not only) in the Cerrado formations of central Brazil. This latter pattern has a closer resemblance to the Atlantic Forest tiger-cat, but nevertheless tends toward yellowish not brownish tone. Melanism has ever been observed only in five specimens, four of which showed a pseudo-melanistic or “platinum” appearance, all in the Cerrado savannas (Supplementary Fig. S15), and one in Eastern Amazonia

with the standard black pattern. The venter is whitish, sometimes with a slightly light grayish or very pale yellowish tone. The tail has the typical large blotch bands, with thin-rings very rarely forming. The species presents two pairs of mammae/teats and ranges in the various savanna formations of the Amazon/Guianas, Cerrado, and Caatinga.

Remarks

As shown, all three species have much intraspecific variation. This extent of variation in the rosette patterns can make some individuals from one species look remarkably like another species (Supplementary Fig. S16). Interesting patterns arise for all of them. For *L. tigrinus* there seems to be a pattern more typical, but not all-inclusive, and restricted to the Guiana Shield and eastern Amazonia (see Supplementary Fig. S5). For *L. guttulus* there is the appearance of the "*tigrinus* pattern" in several specimens of Santa Catarina, as well as in the southernmost limits, in the contact zone with *L. geoffroyi* (Supplementary Fig. S16). For the latter, we suspect this could be a consequence of the bidirectional introgression between *L. guttulus* and *L. geoffroyi* [6], but could it get as far up as Santa Catarina State, and why only in this eastern pocket? It is uncommon, but eventually isolated specimens of either *L. tigrinus* or *L. guttulus* can show a great deal of resemblance to one another, but not so much as becoming common in any region. The "*guttulus* pattern" in *L. tigrinus* occurs in the isolated individuals in the Cerrado northern/central/western savannas, and could even possibly venture into the Pantanal, given the claims of tiger-cats being spotted there (but never photographed; [7]) (Supplementary Fig. S16). The appearance of anomalous/different variations in pelage are mentioned for several cat species (for some more than for others), with the "king-cheetah" being the classical example [8]. One very important issue to keep in mind is, skin/color/spots can vary (even a lot), but the body patterns only rarely do so. The "*pardinoides* cloudy pattern" has only been seen on specimens of *L. guttulus*. As mentioned before, specimens of *L. pardinoides* from the southern Andes, from the Peruvian Yungas all the way to NW-Argentina, consistently show a distinguished rosette pattern, rounder and larger that do not coalesce or tend to (similar to those of *guttulus*, but larger). This pattern is distinctive from that of NW-Andes (Colombia, Venezuela, Ecuador). More intriguingly, the NW-Andean pattern of *L. pardinoides pardinoides* is very similar to that of *L. pardinoides oncilla* of Central America and distinct from the supposedly same subspecies/population found in the southern Andean range (Peru, Bolivia, Argentina). Could this

be an indication of a distinct taxonomic entity: perhaps, a subspecies for the southern Yungas? Bonilla-Sánchez [9] mentions of a possible barrier between N- and S-Andes at the Huancabamba Depression. Other medium-large-sized mammals, such as the mountain coati (*Nasuella olivacea*), the Andean tapir (*Tapirus pinchaque*), the olinguito (*Bassaricyon neblina*), and the dwarf red brocket deer (*Mazama rufina*), are known to only range in the NW-Andes, either north or just slightly south of the Huancabamba [10]. This could possibly justify the differences. However, detailed genetic analysis would be needed to sort this out.

Indeed, the similarities go above the tiger cat level into spotted sympatric species of *Leopardus*. They are easily confused and the misidentification of *Leopardus* species is very common [11–13]. Photographic, morphological, and skin pattern portrayals of the complex in books and guides usually mix them up and do not provide clear and accurate/proper depictions or portrayals.

Diagnostic Differences from other Sympatric *Leopardus*

Externally, all tiger-cats can be differentiated from margays (*Leopardus wiedii*) and ocelots (*Leopardus pardalis*), by the presence of a lateral convex head profile and nape hairs not reversed, i.e., slanting backward from head to tail base. There is closer resemblance to the margay than to the much larger ocelot and with the two species they hybridize with, Geoffroy's cat and Pampas cat, or any other felid of *Leopardus*. From the margay, tiger-cats also differ by having, on average, smaller head and body length, a slightly smaller tail, whereas margays have proportionally large paws and bulging eyes [11]. A common mistake is the assumption that only margays have a long tail. Both savanna and clouded tiger-cats (and a few Atlantic Forest tiger-cat) frequently have long margayish-sized tails. The Atlantic Forest/southern tiger-cat differs from Geoffroy's cat as it does not have the spot pattern of small solid dots, usually grouped by two on the sides, but not forming rosettes. Geoffroy's cats are also larger, have a bulkier build, and are heavier (3.7 kg at their border area vs. 2.3 kg of *L. guttulus*) with a shorter tail [10,11,14]. Brazilian Pampas cats (*Leopardus braccatus*) have stripes on limbs, pointed ears, long hair, shorter tail, and typically in Brazil unspotted coat pattern [10,11]– Supplementary Fig. S17. The hybrids, that are partly *geoffroyi* partly *guttulus*, *geoffruttulus*, or *guttoffroyi* (depending on where most resemblance is), such that cannot be decided to which group it would be, are larger, bulkier and with heavy head and shorter tail. One live-trapped specimen had HB = 770 mm, tail = 190 mm (abnormally short), ears = 45 mm, and body mass of 3.6 kg (Supplementary Fig. S18).

Cranially, tiger-cat species differ from other *Leopardus* by the much less convex dorsal outline with a flatter frontal area (that is only slightly arched), by the narrower braincase, shorter and slender postorbital processes, and a less inflated and narrower auditory bullae in proportion to its length in the species complex [3]. The differences in the length of the upper carnassial in the tiger-cat complex and in the margay confirms the rule of Husson [15] that PM4 <10 mm for tiger-cats, and larger than that for margay. However, it should be corrected to PM4 <11 mm for all species of tiger-cats and is higher than that for the margay. In this way, the upper canine diameter and the upper carnassial length could be considered diagnostic cranial features of all tiger-cat species from the other Neotropical felids.

Taxonomic Notes

The taxonomic scheme proposed here is different from all available: it places *pardinoides* and *oncilla* together and elevates the group as *pardinoides* to the species level, separate from *L. tigrinus* and *L. guttulus*. The current arrangement of Kitchener et al. [2] of IUCN/SSC/Cat Specialist Group, taking into account the morphological, molecular, and biogeography considers *L. guttulus* as a full species and *L. tigrinus* encompassing *tigrinus, pardinoides*, and *oncilla*. Another scheme proposed by Nascimento and Feijó [16], which also considers *L. guttulus* as a full species and separates the northeastern Brazilian population as *Leopardus emiliae* (Thomas, 1914), from that of the Guianas, which was considered alongside *pardinoides* and *oncilla*, all under *L. tigrinus*. Other than Nascimento and Feijó [16], no other taxonomic scheme has considered *L. emiliae* as a proper species or a distinct subspecies. Although we did not conduct craniodental analysis, our approach used a combination of morphologic, phenotypic, and ecological modeling. Kitchener et al.'s [2] review showed a level of uncertainty, only regarding *pardinoides* as a subspecies or species, owing to the lack of molecular data, considering it "possibly distinct or even a distinct species," and that *oncilla* might also be distinct. This uncertainty is no longer existing [17]. Additionally, de Oliveira et al. [18] showed that the skin patterns (one of the attributes considered by Nascimento & Feijó [16]) of the Guiana specimens were completely distinct from those of northwest South America (i.e., *pardinoides*) and those of Central America (i.e., *oncilla*) and with very similar patterns to those of Brazil. Our perception aligns with Cabrera's [19] view, which considered *Felis (Leopardus) tigrina tigrina* from northeastern Brazil to E-Venezuela, i.e., placing "*L. emiliae*" within this group (as a synonym).

Thus, we maintain the original grouping, where the Guianas and Brazil are kept under the subspecies *L.t. tigrinus*, which is now a species without any previously established subspecies, until genetic proof is provided showing them to be distinct at the species or subspecies level. Therefore, our scheme invalidates the proposal of *L. emiliae* as a full species by Nascimento and Feijó (2017), which was not previously even regarded as subspecies by any recent mid-20th or 21st century taxonomic arrangement (e.g., [2,19–22]).

It is our understanding that what might have led Nascimento and Feijó [16] to place the Guiana Shield specimens alongside *pardinoides* and *oncilla* was the lack of samples with coalescing spot patterns forming small–medium-sized oblique bands, which they mention do not coalesce for the northeastern Brazilian specimens, which is not the case. We have several photographic records of specimens from the Cerrado and Caatinga (Supplementary Fig. S13), as well as the border area with the Amazon in northeast, central, and central-western Brazil showing the same patterns as found in the Guianas and NE-Venezuela (Supplementary Figs. S3, S4). The advent of camera trapping has allowed us to see a much broader range of skin patterns, including felids. As such, photographs also supporting the museum skins, which usually date from the late 19th to the mid-20th centuries, of the small Neotropical *Leopardus* to show a much broader range and patterns of coat variation, including different spot patterns, melanism, leucism, etc. [23–25]. Additionally, they provide a 3D view of the specimen that is not seen in a flat museum skim.

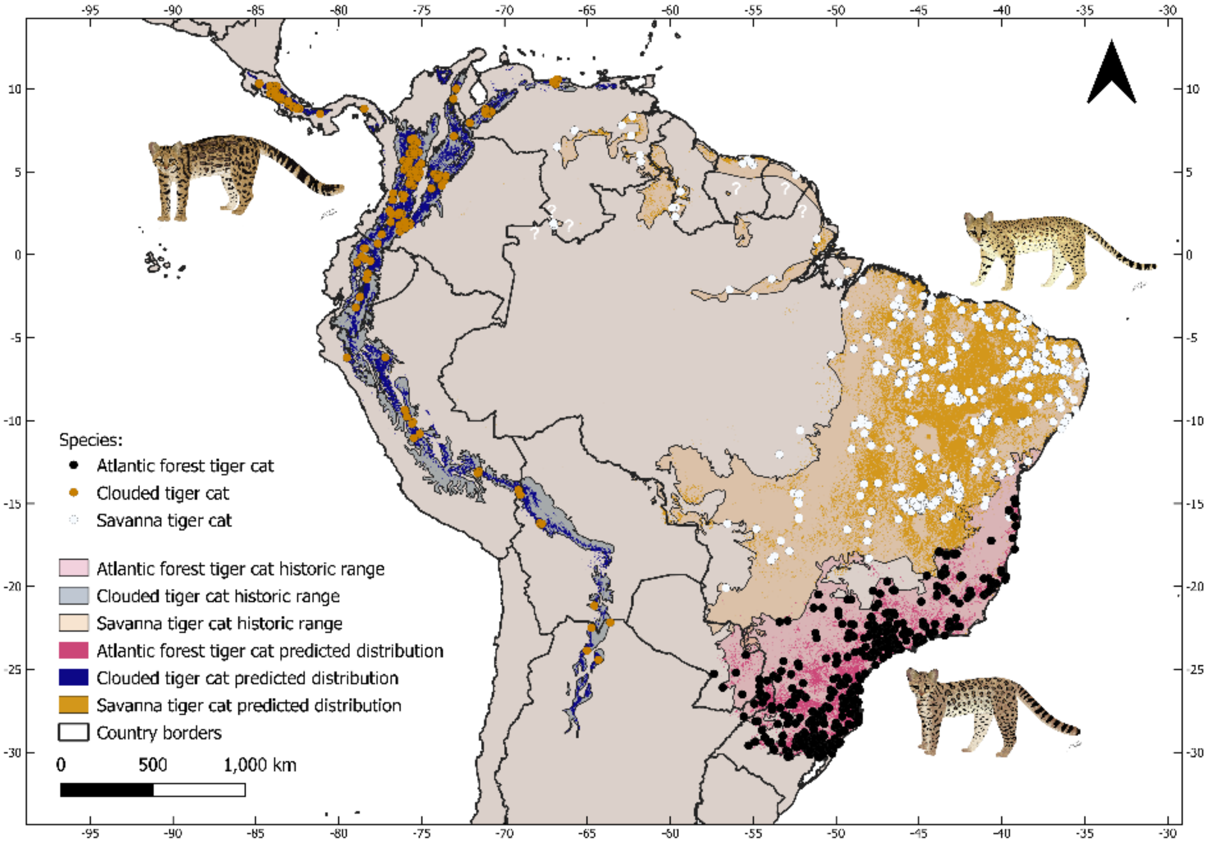

Figure S1. Locations of the 1,439 record points of all tiger-cats across their historic and current ranges. Figure made on QGIS v. 3.28.12 LTR (www.qgis.org).

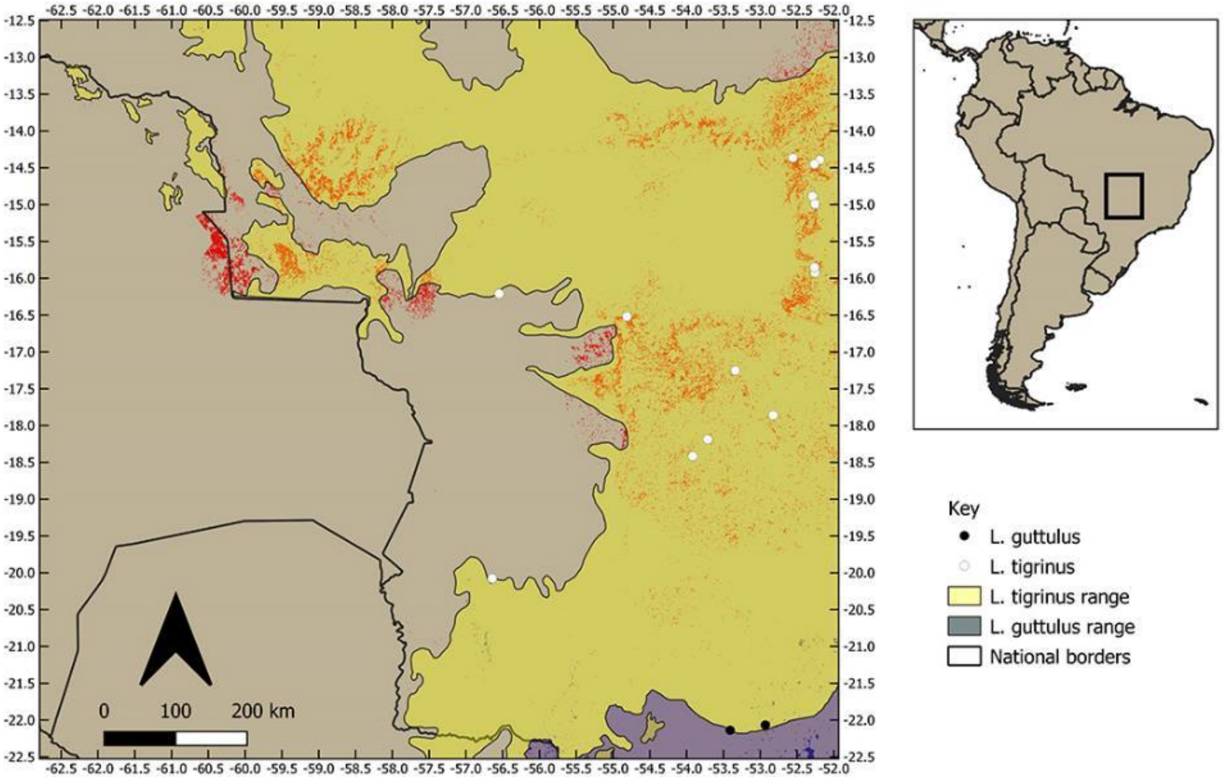

Figure S2. Tiger-cat records closest to the Pantanal: there are records of savanna tiger-cats (*L. tigrinus*) at the borders of the Pantanal, whereas the closest records of Atlantic Forest tiger-cats (*L. guttulus*) are much further away, at the Cerrado/Atlantic Forest border area. Figure made on QGIS v. 3.28.12 LTR (www.qgis.org).

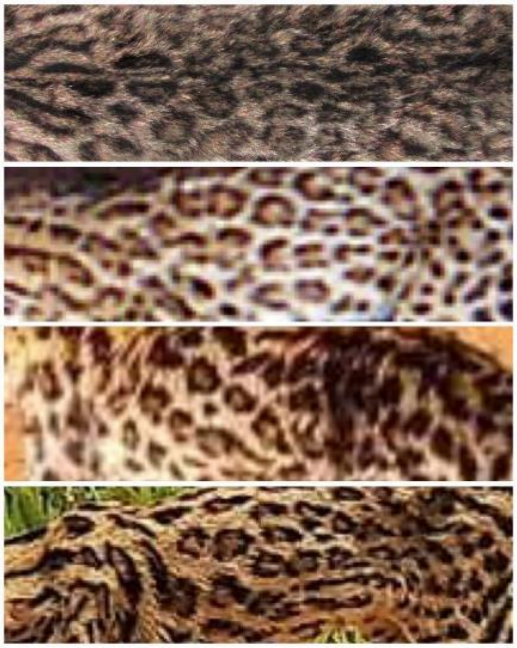

Figure S3. The *guttulus*-like patterns in tigrinus specimens: skin patterns from the alleged specimen from the Pantanal (top) and those of live *Leopardus tigrinus* from other savanna areas of central and central-western Brazil, showing more *guttulus*-like than *tigrinus*-like rosettes — authors.

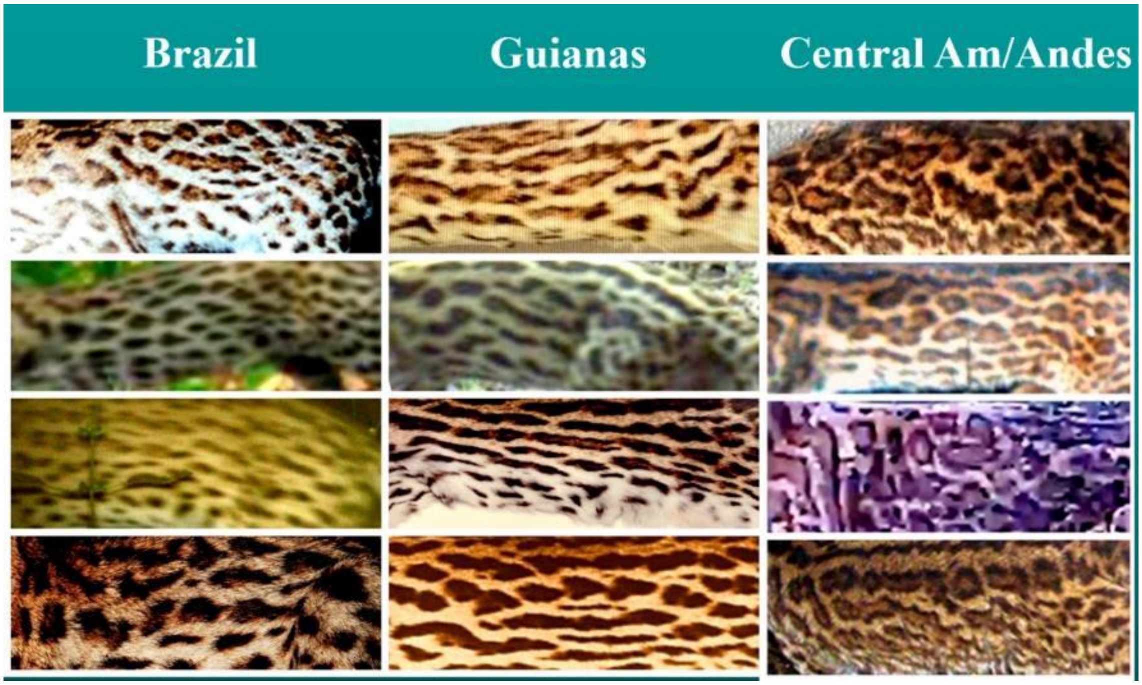

Figure S4. Skin patterns of Northern tiger cat specimens from the Guianas compared with those from Brazil, Central America, and the tropical Andes (de Oliveira et al. (9).

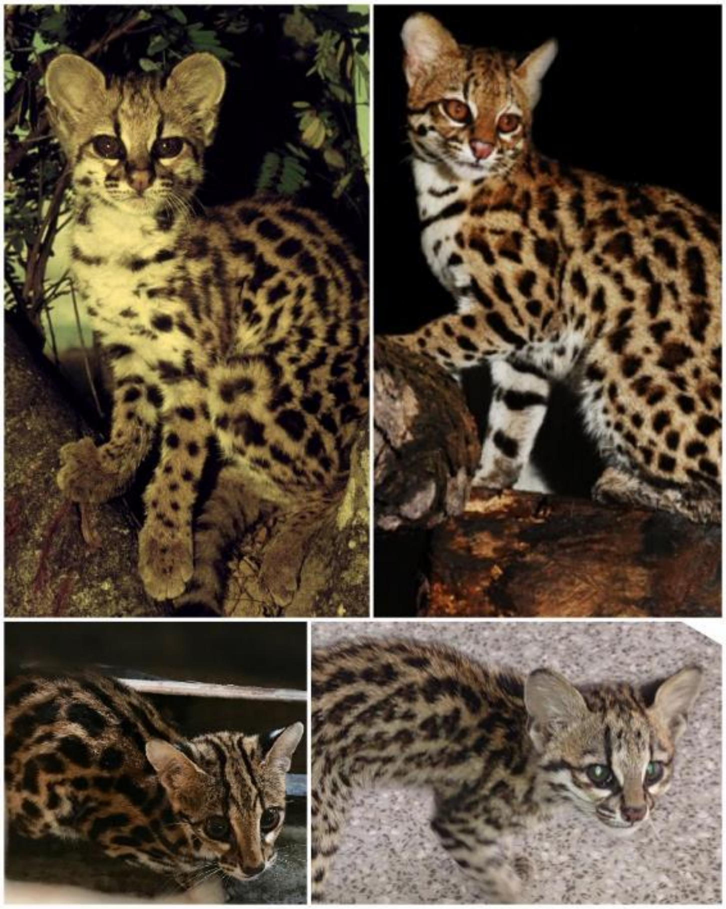

Figure S5. Guiana Shield—Brazil resemblances: specimens of the Guiana Shield area (left) and those of Eastern Amazonia in Brazil (right). Note that only the top right specimen is an adult. Photo credits — Top Rafael Hoogesteijn; bottom Amanda Moreira.

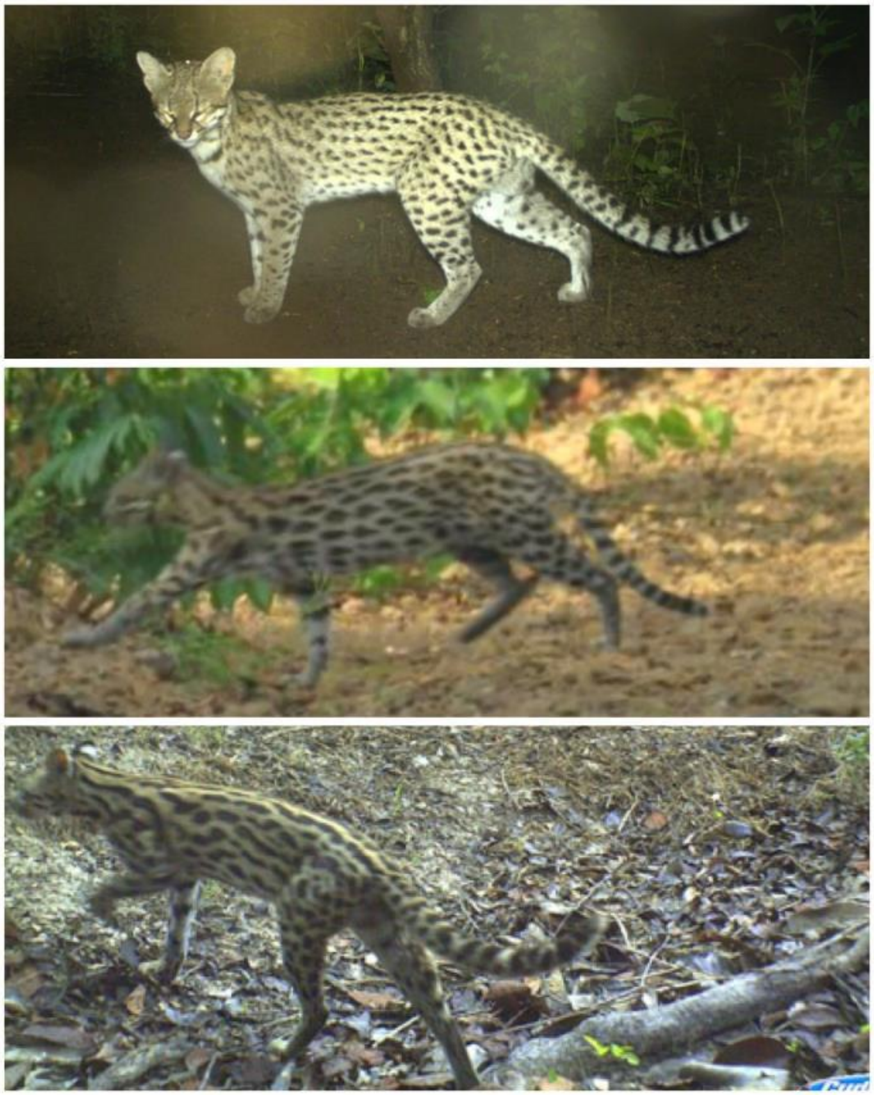

Figure S6. Detecting repeated patterns on camera: camera trap pictures of *Leopardus tigrinus* specimens from the semi-arid Caatinga shrub-woodland (authors/Wild Cats Americas Conservation Program), from the central savanna of Brazil (Gilson Afonso Júnior), and from the Rupununi savanna in Guyana (Evi Paemelaere). Note the same body pattern of skinny-looking, long-legged, very long thin tails, large ears, with varying degrees of solid dot-like rosettes in a pale yellowish background.

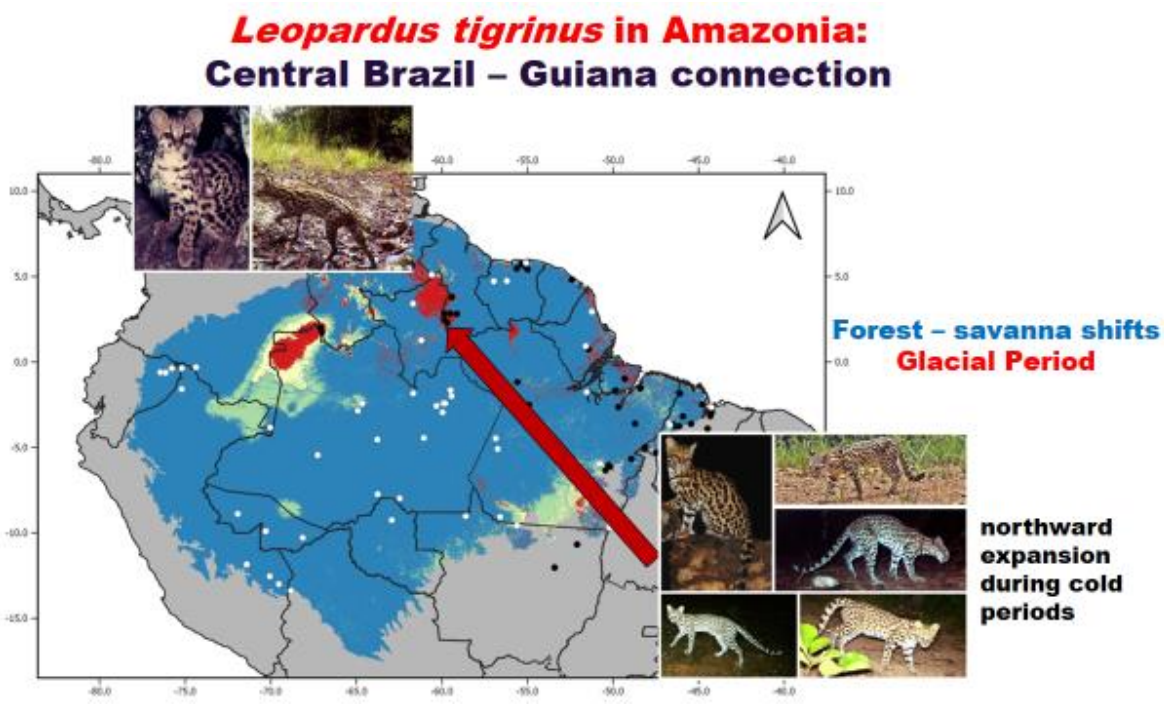

Figure S7. The Pleistocene colonization: the savanna tiger-cat (*Leopardus tigrinus*) likely reached the Guiana Shield during the forest–savanna shifts of the glacial period, with a northward expansion during cold periods (adapted from de Oliveira et al. [18]).

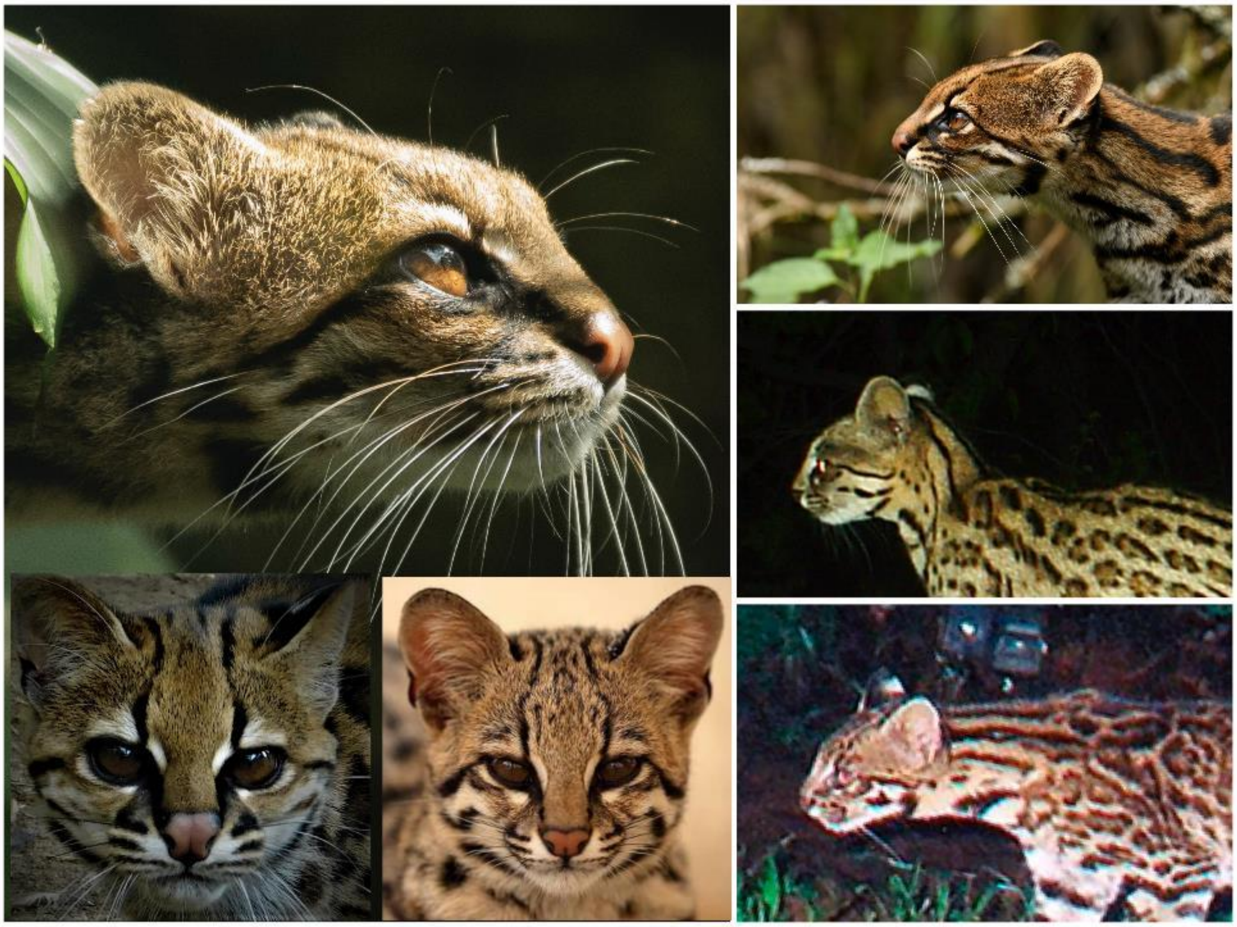

Figure S8. Head profiles: all tiger-cats have the diagnostic characteristics of the nape hairs slanting backward and a convex head profile. Right side (top to bottom): *L. pardinoides*, *L. tigrinus*, *L. guttulus*. Photo credits — Top left Gilder Esteban González-Montenegro, top right Johannes Pfleiderer; all other are from authors.

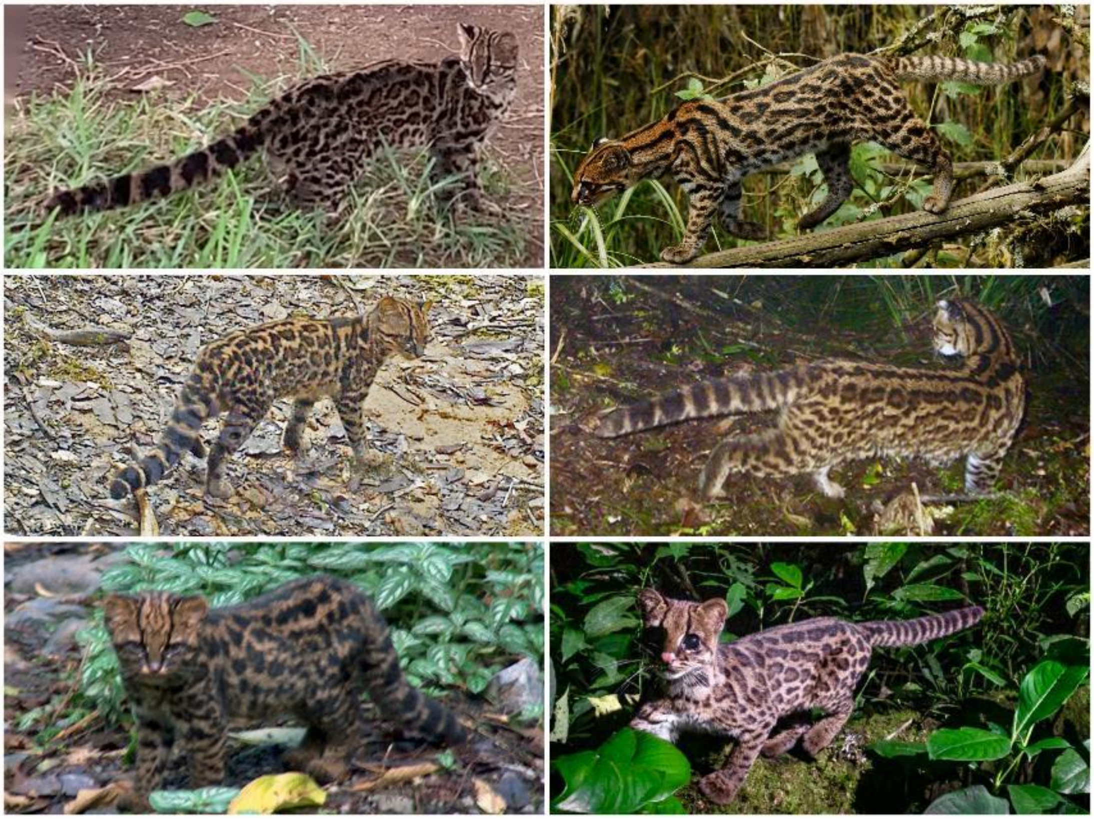

a

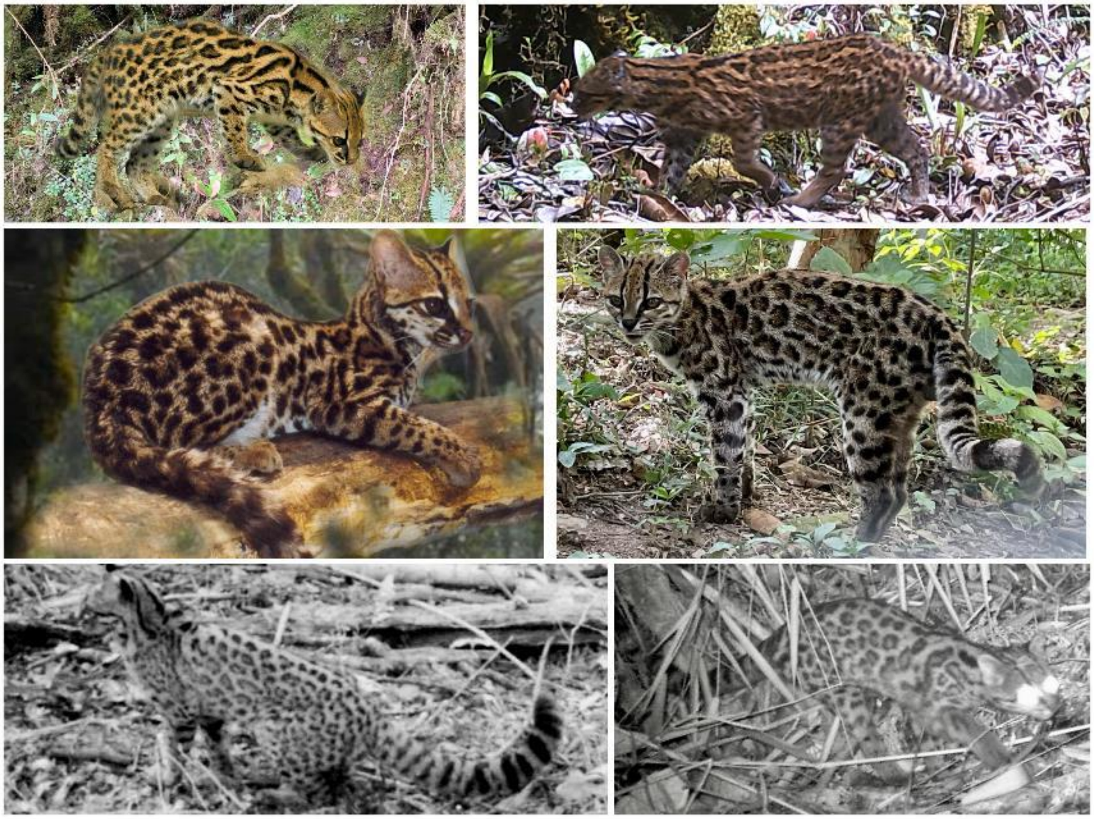

b

Figure S9. *Leopardus pardinoides*: a) Specimens of clouded tiger-cat (*L. pardinoides*) from Central America (*L. p. oncilla*) and the northern Andes (*L. p. pardinoides*), note the similarity among them; b) Northern Andean specimens (top pictures) and those of the southern Andes Yungas, note the different patterns between them, possibly indicative of a new taxonomic unit (subspecies)? Photo credits — a) top right Johannes Pfleiderer, bottom right Camilo Botero; b) middle right Paola Nogales-Ascarrunz, bottom right Guido Ayala, bottom left Roberto Cáceres; all other pictures (a, b) are from authors.

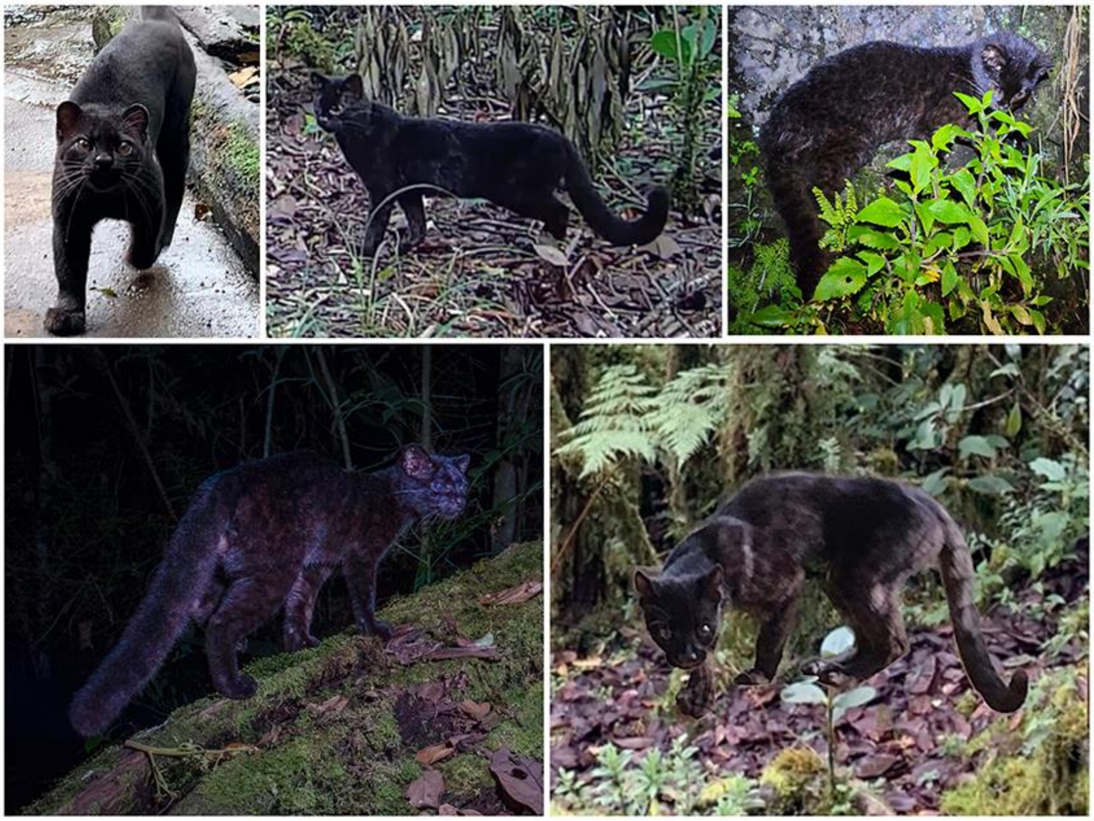

Figure S10. Melanistic specimens of the clouded tiger-cat (*L. pardinoides*) from Central America, Northern and Southern Andes. In six areas of Costa Rica, melanistic records accounted for 32%, whereas in areas of Ecuador it was 18.4%, and 6.5% in four sites in the middle Cauca of central-western Colombia. Photo credits — top right Gabriel Archondo, bottom left Benjamin Luke, all other pictures are from authors.

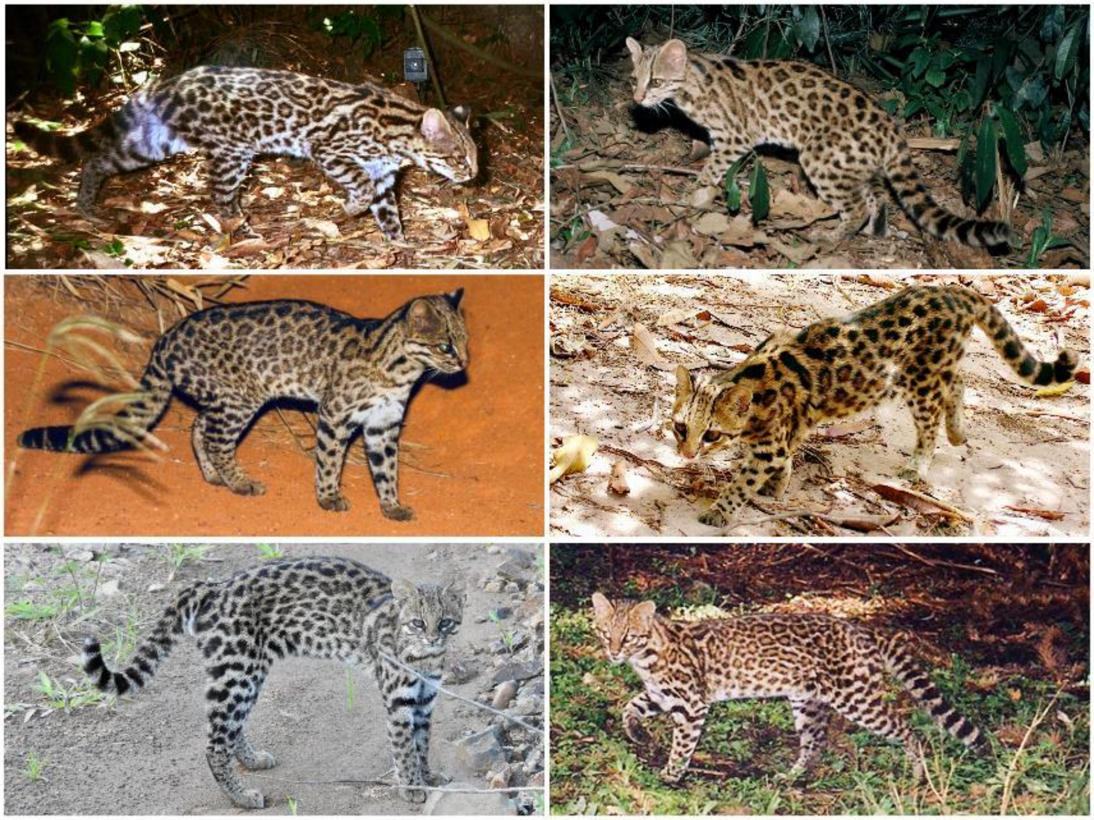

a

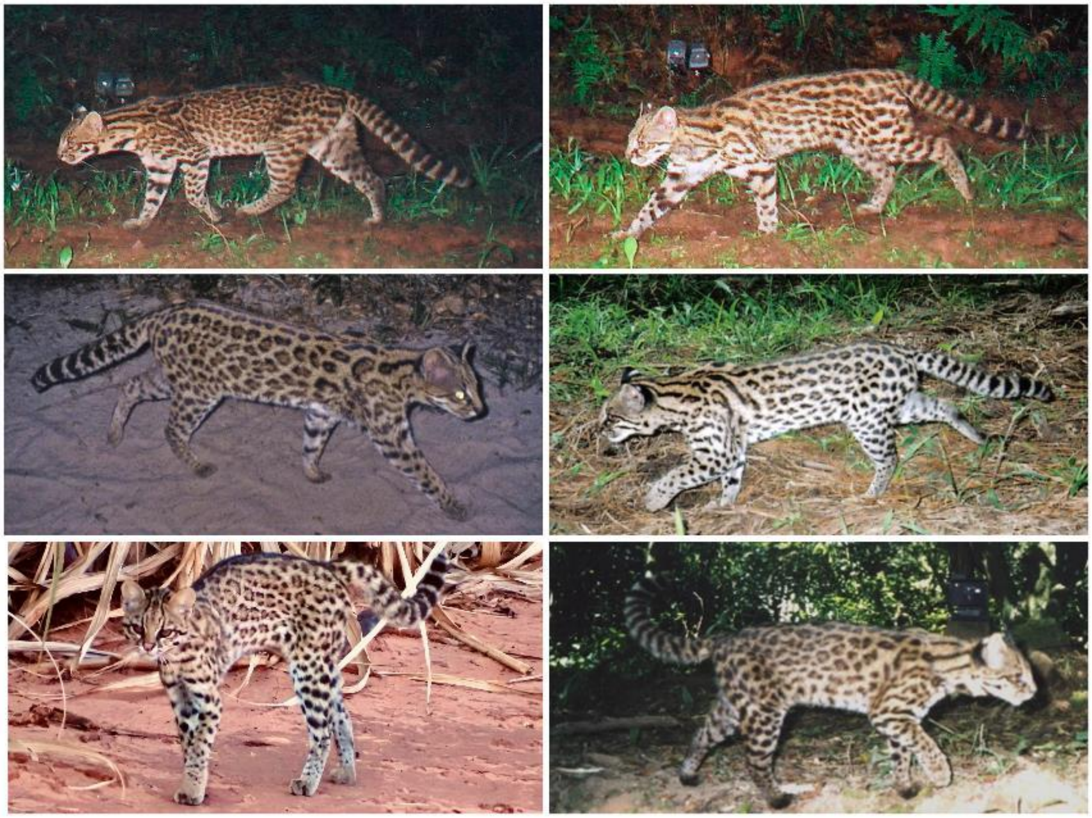

b

Figure S11. Skin patterns of the Atlantic Forest tiger-cat (*L. guttulus*) from across its range. Photo credits — a) middle left Gustavo Pinto, bottom left Roberto Tomasi Jr.; b) middle right Fernando Tortato, bottom left Weslley Alves; all other pictures (a, b) are from authors.

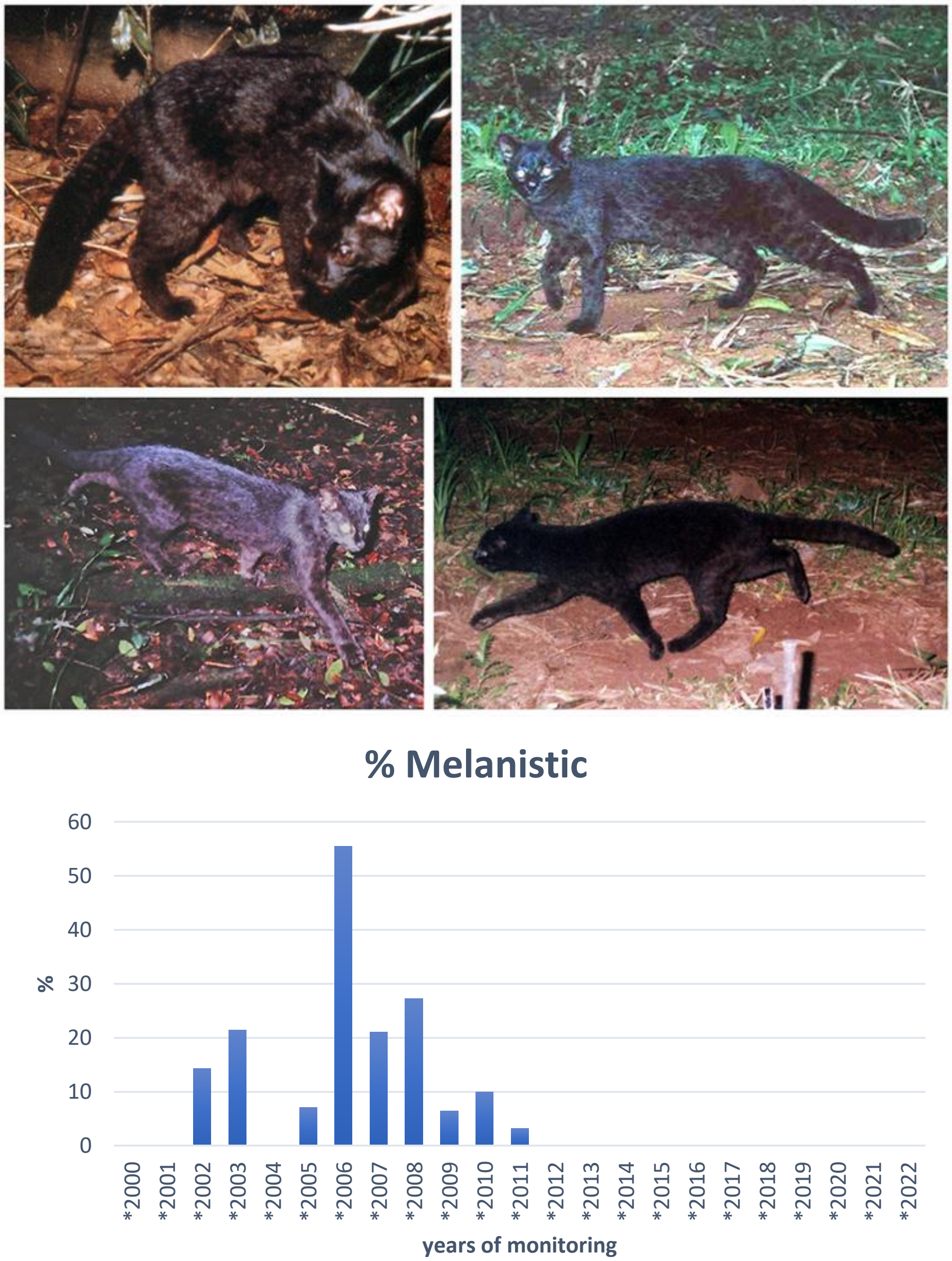

Figure. S12. Melanistic specimens of the Atlantic Forest tiger-cat (*L. guttulus*). Of the total records, melanistic individuals accounted for 0% in Itapeva State Park (9-year period), 2.5% in the Aguaí Biological Reserve area (5-year period), and 18.1% in Caraguatá and Serra do Tabuleiro/SC (4-year period, Graipel et al., 2014). Only in the southernmost area of the species range, in the Taquari Valley, we recorded 8.11% in a 2-year period. Over a 23-year period, the percentage of melanistic records at São Francisco de Paula National Forest was on average 7.2% ± 13.4%, but showed dramatic fluctuations, ranging from 0% to 55.6%; more intriguingly, since 2012 up to 2022, there has been no record of any melanistic specimens. Photo credits — Bottom left Marcelo Mazzolli, all others are from authors.

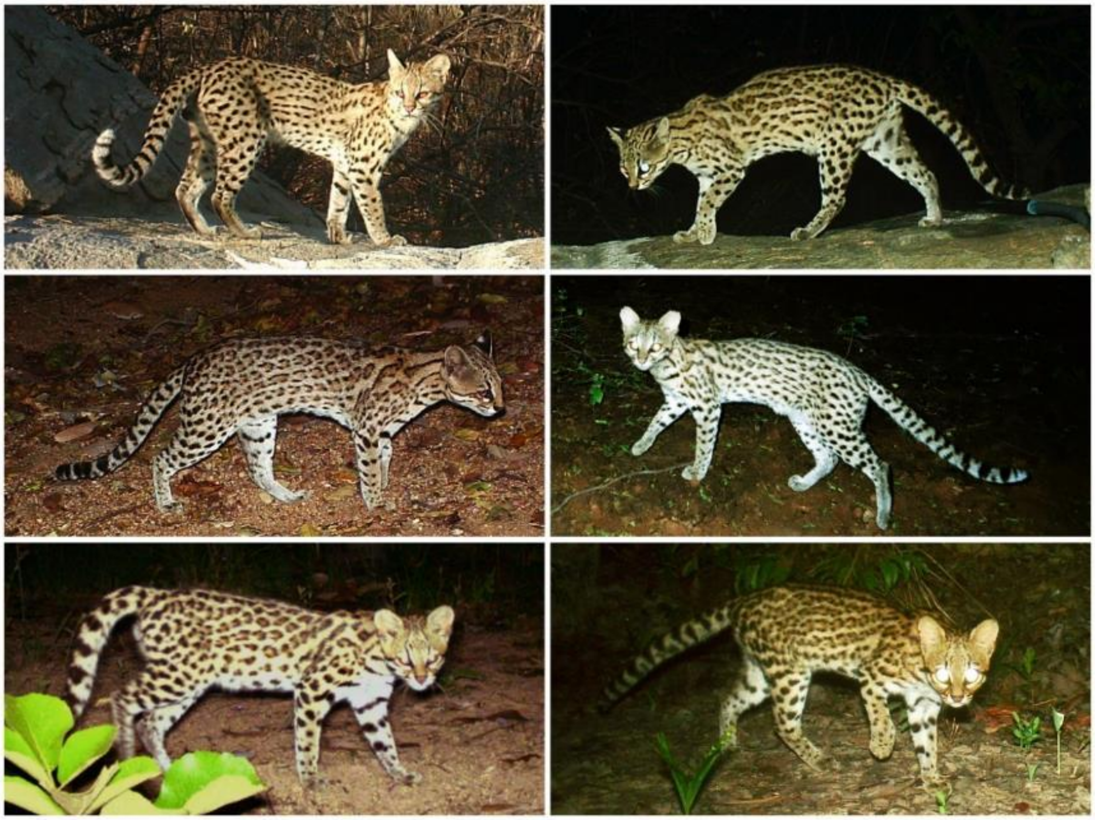

a

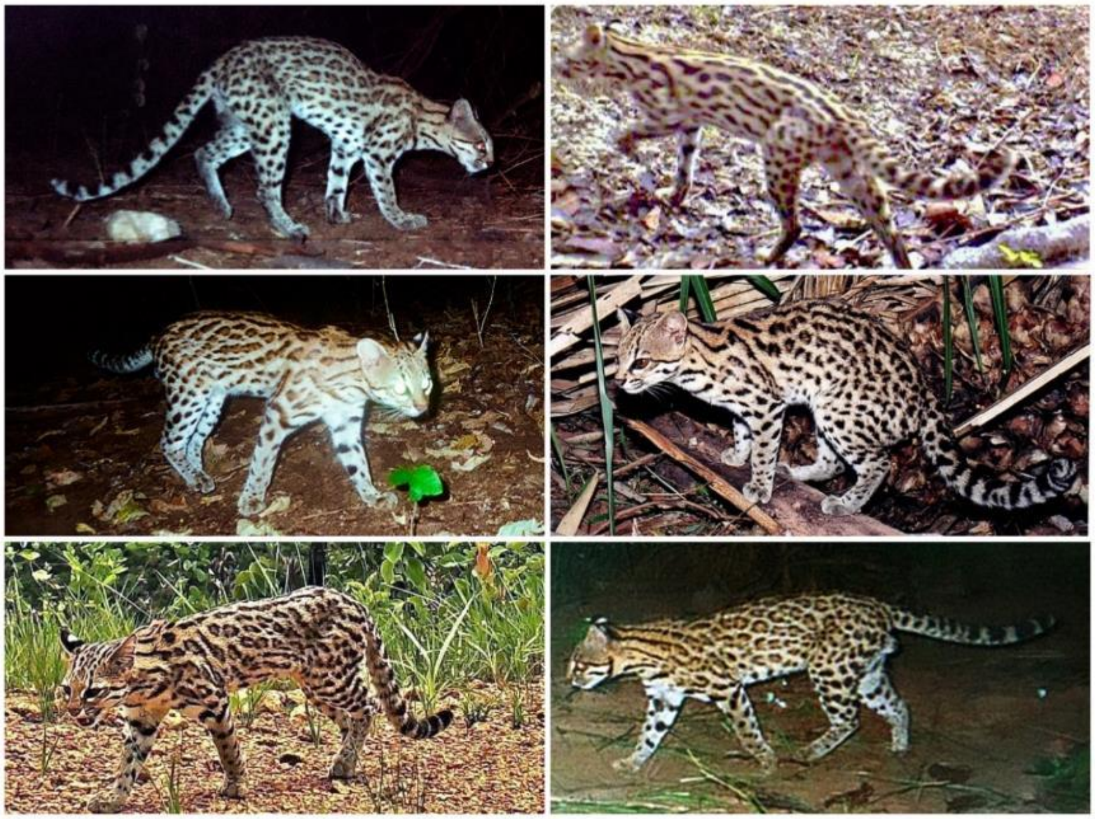

b

Figure S13 a, b. The variety of skin patterns of specimens of savanna tiger-cat (*L. tigrinus*) from the entire range: Guiana Shield, eastern Amazonia, northern and central savannas, semi-arid Caatinga woodland/shrubland. Photo credits — b) top right Evi Paemelaere, bottom left Fábio Hudson S. Soares/Brasília é o Bicho; all other pictures (a, b) are from authors

.

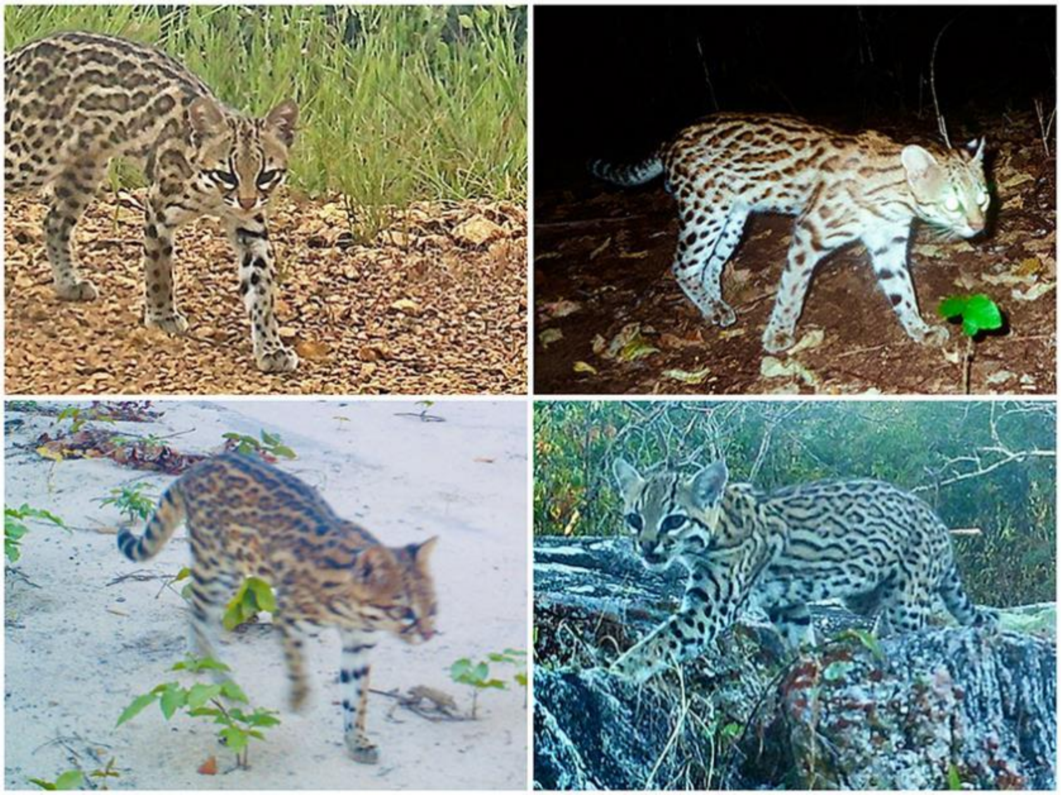

Figure S14. Ocelot spots on savanna tiger-cats (*L. tigrinus*) from central and northeastern Brazil showing the coalescing oblique spots, such as those of ocelots (*L. pardalis*) that are also found on clouded tiger-cats (*L. pardinoides*). Photo credits — top left Fábio Hudson S. Soares/Brasília é o Bicho, bottom right Bruno Bezerra; all other are from authors.

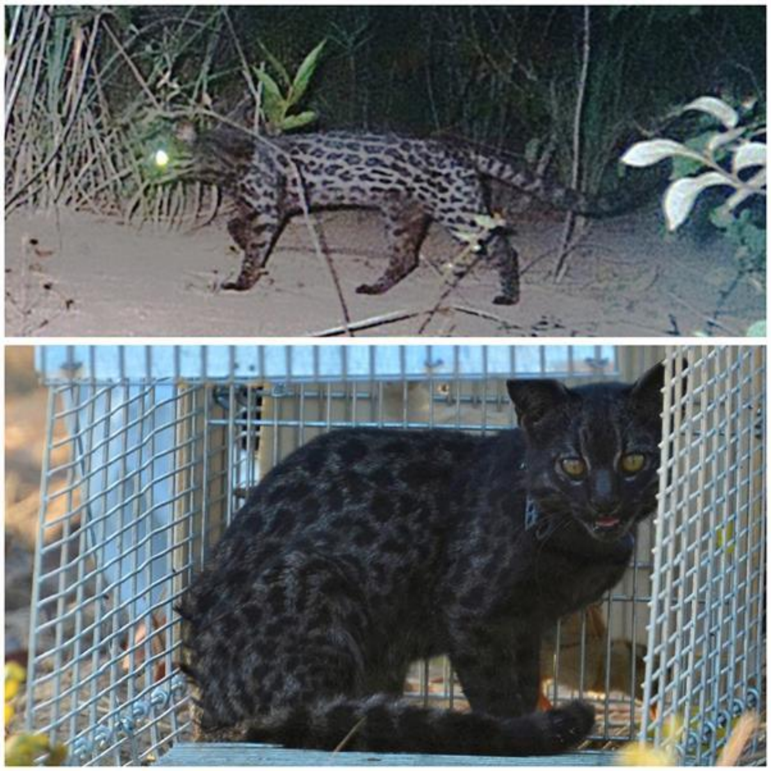

Figure S15. Melanistic specimens of the savanna tiger-cat (*L. tigrinus*). Only five specimens have ever been recorded, four of which displayed this “pseudomelanistic/ platinum” pattern. Photo credits — authors.

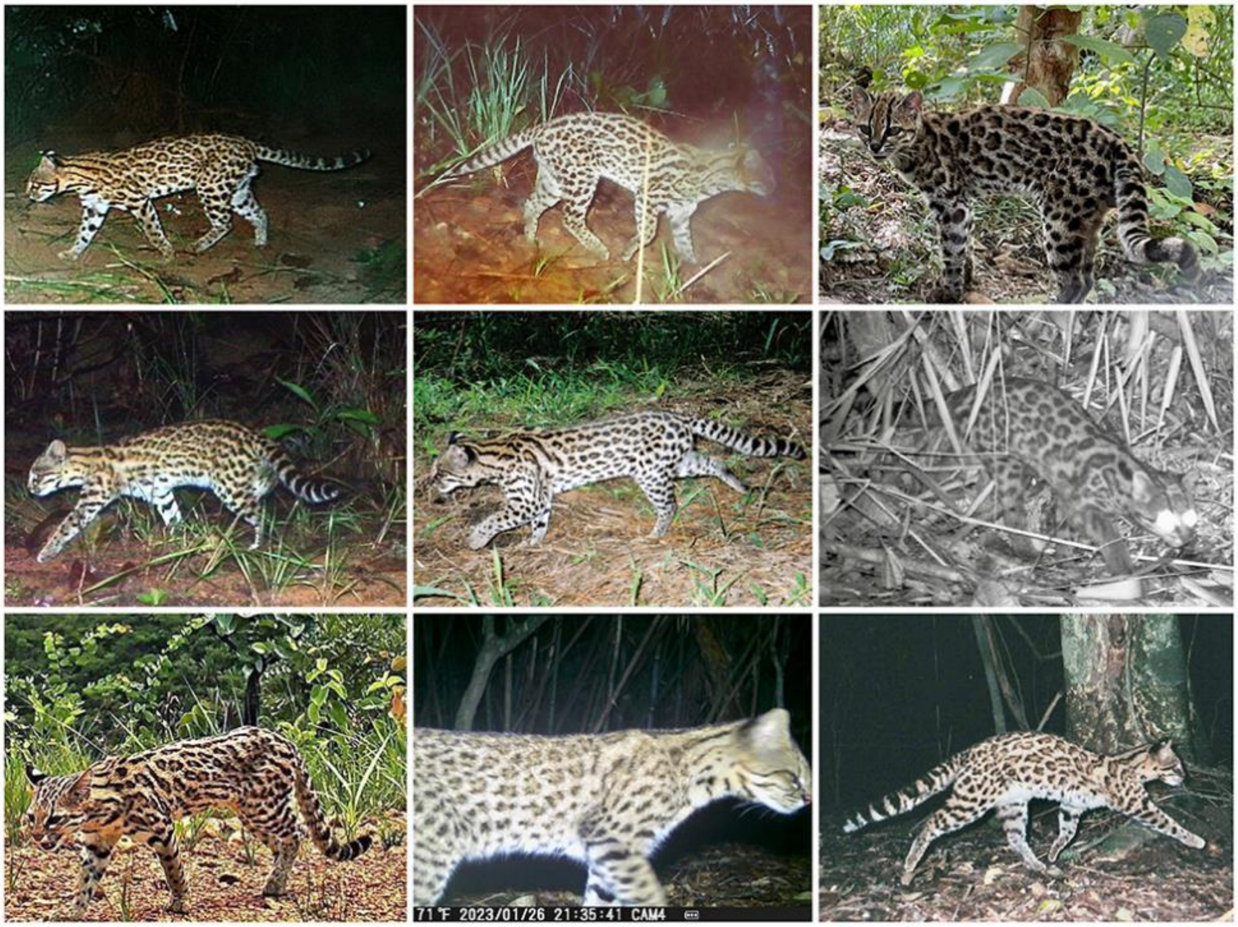

Figure S16. “Wearing” each other’s coats: *Leopardus tigrinus* showing the *L. guttulus* coat pattern (left column); *Leopardus guttulus* wearing the *L. tigrinus* coat (the middle photo is from the area of Santa Catarina where this pattern is common, and the bottom picture is from the contact zone with *L. geoffroyi*); and Southern Andean *Leopardus pardinoides* showing the *L. guttulus* pattern (right column, top 2), and *L. guttulus* showing the cloudy-like spots of *L. pardinoides* (lower right). Photo credits — First column lower picture Fábio Hudson S. Soares/Brasília é o Bicho; Middle column middle picture Fernando Tortato; Right column top picture Paola Nogales-Ascarrunz, middle picture Guido Ayala; all other pictures are from authors.

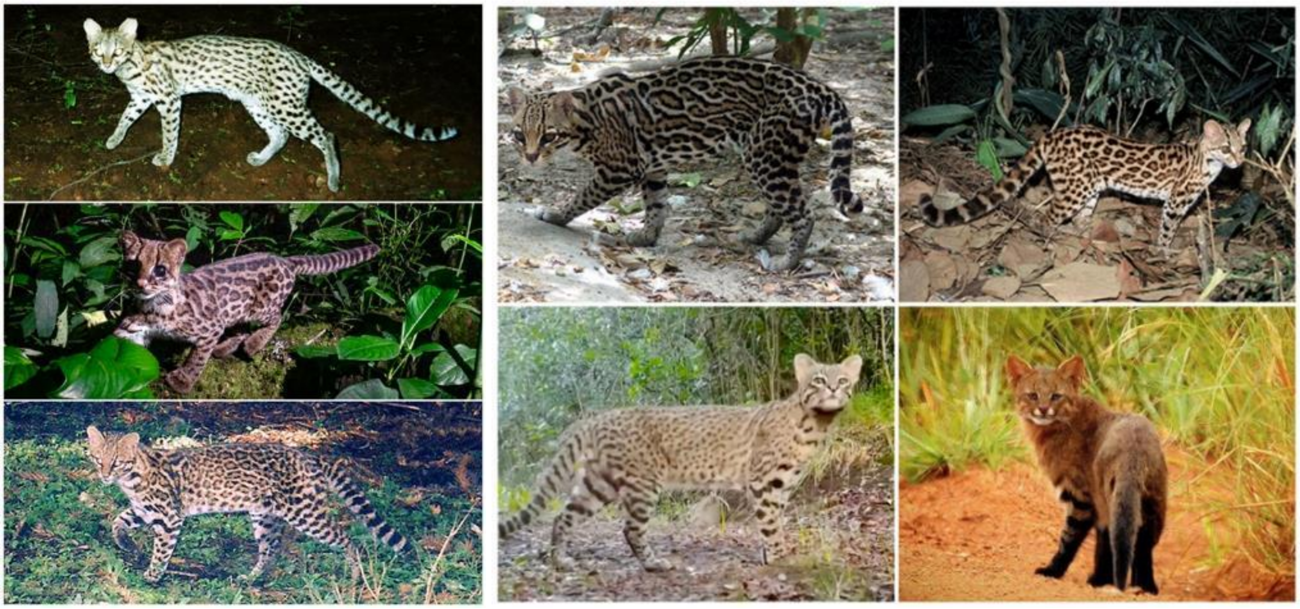

Figure S17. The tiger cat species complex (*L. tigrinus, L. pardinoides, L. guttulus*) and the other sympatric *Leopardus* species (*L. pardalis*, ocelot, *L. wiedii*, margay, *L. geoffroyi*, Geoffroy's cat, and *L. braccatus*, Brazilian Pampa cat). Photo credits — First column middle picture Camilo Botero; 3rd column lower picture Kennedy Borges; all other pictures are from authors.

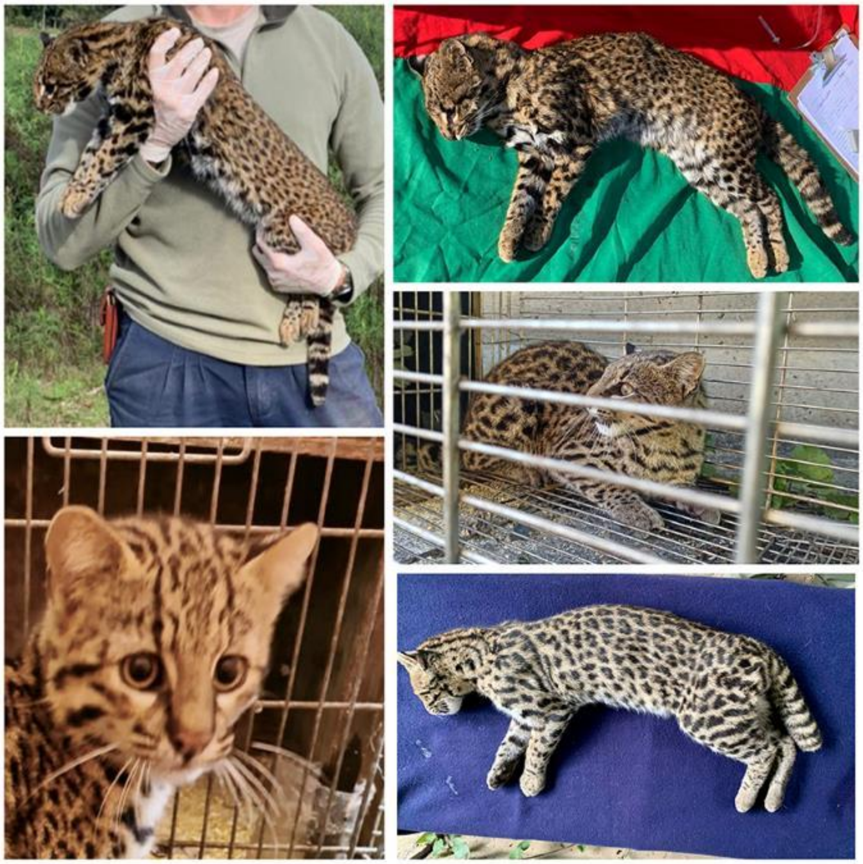

Figure S18. A *geoffroyi/guttulus* hybrid, "*geoffruttulus*". Photo credits — authors.

Table S1. ANOVA results for environmental variables among species of the *Leopardus tigrinus* complex.

Environmental variable	F	P value
Elevation	2503.7	<0.001
Terrain Ruggedness Index	734.87	<0.001
Net Primary Production	913	<0.001
Gross Primary Production	582.43	<0.001
Canopy Height	479.79	<0.001
Tree cover	310.35	<0.001
Annual Mean Temperature	1880.2	<0.001
Mean Diurnal Range	152.16	<0.001
Isothermaility	2377.3	<0.001
Temperature Seasonality	2627.1	<0.001
Max. Temperature of Warmest Month	2153.4	<0.001
Min. Temperature of Coldest Month	1021.2	<0.001
Temperature Annual Range	768.46	<0.001
Mean Temperature of Wettest Quarter	1575.5	<0.001
Mean Temperature of Driest Quarter	1391.5	<0.001
Mean Temperature of Warmest Quarter	2082.3	<0.001
Mean Temperature of Coldest Quarter	1603	<0.001
Annual Precipitation	616.61	<0.001
Precipitation of Wettest Month	235.51	<0.001
Precipitation of Driest Month	652.32	<0.001
Precipitation Seasonality	847.3	<0.001
Precipitation of Wettest Quarter	212.37	<0.001
Precipitation of Driest Quarter	692.73	<0.001
Precipitation of Warmest Quarter	836.73	<0.001
Precipitation of Coldest Quarter	267.76	<0.001

Table S2. Tiger-cat range per country in km^2, with percentage of species range in parenthesis.

Country	*L. pardinoides*	*L. tigrinus*	*L. guttulus*
Costa Rica	17,009 (4.15%)		
Panama	11,119 (2.72%)		
Venezuela	23,202 (6.4%)	15,623 (1.05%)	
Colombia	162,214 (39.63%)		
Ecuador	46,824 (11.44%)		
Peru	84,870 (20.73%)		
Bolivia	38,225 (9.34)		
Paraguay			11,563 (2.90%)
Argentina	22,908 (5.60%)		16,823 (4.20%)
Brazil		1,468,806 (98.59%)	371,218 (92.90%)
Guyana		8,036 (0.54%)	
Suriname		6,346 (0.43%)	
French Guiana		49 (0.00%)	

Table S3. Details on some body and dental measurements of the tiger-cat complex, *Leopardus pardinoides*, *Leopardus tigrinus*, and *Leopardus guttulus*, and comparisons with *Leopardus wiedii* head and body/tail proportions and dental metrics used for the statistical analyses.

**Parameter**	***L. pardinoides***	***L. tigrinus***	***L. guttulus***	***L. wiedii***
Head & Body – HB (mm)				
mean	484.86	481.52	509.61	
standard dev.	30.98	56.32	33.91	
median	488	433	510	
min	410	380	449	
max	540	650	591	
N	21	21	44	
Tail Length – TL (mm)				
mean	290.43	278.05	271.8	
standard dev.	28.86	31.71	21.44	
median	290	280	270	
min	245	210	212	
max	340	345	310	
N	21	20	45	
Ear (mm)				
mean	38.6	42.56	41.63	
standard dev.	5.35	4.64	5.62	
median	40	41	42.5	
min	30	36	30	
max	45	50	51	
N	10	18	23	
Body Mass (kg)				
mean	2.27	2.32	2.35	
standard dev.	0.464	0.400	0.451	
median	2.20	2.19	2.34	
min	1.8	1.85	1.1	
max	3.4	3.46	3.47	
N	10	22	46	
TL/HB				
mean	0.6	0.578	0.534	0.659
standard dev.	0.053	0.053	0.039	0.065
median	0.583	0.571	0.543	0.638
min	0.52	0.495	0.434	0.564
max	0.695	0.682	0.596	0.791
N	21	20	44	32

E/HB (%)				
mean	7.764	9.076	8.118	
standard dev.	1.218	1.168	1.06	
median	8.095	9.1	8.3	
min	5.6	6.5	5.9	
max	9.2	10.9	10.3	
N	10	17	22	
Upper canine diameter (mm)				
mean	4.238	4.058	4.051	
standard dev.	0.353	0.303	0.426	
median	4.33	4.05	4.1	
min	3.8	3.5	3	
max	4.6	4.6	5.2	
N	10	10	61	
Canine diameter complex				
mean	4.083			5.241
standard dev.	0.407			0.643
N	78			76
Upper premolar - PM4 complex				
mean	9.98			11.14
standard dev.	0.52			0.77
N	37			62

Table S4. Descriptive statistics of environmental variables used for modeling tiger-cat complex distribution for each species.

**Environmental variable**	***L. pardinoides***			***L. tigrinus***			***L. guttulus***		
	**Mean (±SD)**	**Median**	**Range**	**Mean (±SD)**	**Median**	**Range**	**Mean (±SD)**	**Median**	**Range**
Elevation (m)	2364 (±623)	2379	540–3960	495 (±275)	497	3–1254	552 (±397)	585	0–1817
Terrain Ruggedness (m)	482 (±243)	460	31–3000	77.5 (±76)	52	2.2–711	160 (±152)	100	4.69–1033
Net Primary Production (kgC/m^2)	10773 (±3821)	10391	977–23263	5810 (±2211)	5528	280–15333	13937 (±3589)	14250	4456–18399
Gross Primary Production (kgC/m^2)	22063 (±5784)	23647	4385–36784	13616 (±4453)	12770	2201–33326	24908 (±6795)	26860	7552–33599
Canopy Height (m)	20 (±13)	20	0 – 103	4.2 (±6.0)	3	0–101	11.7 (±13.2)	12	0–101
Tree cover (%)	82 (±20)	90	0–100	26.7 (±33.3)	4	0–100	60.6 (±42.5)	89	0–100
Annual Mean Temperature (°C)	14.4 (±36)	14.5	5.4–24.6	24.3 (±1.9)	24.2	19.1–27.8	19.0 (±1.9)	19.1	13–24.4
Mean Diurnal Range (° C)	9.5 (±17)	9.1	7.5–15.8	11.4 (±1.7)	11.5	6.5–15	10.5 (±1.63)	10.5	6.5–13.9
Isothermaility (%)	84 (±6.7)	84	53–95	73 (±4.3)	72	62–93	56.4 (±6.7)	56	43–69
Temperature Seasonality (° C)	4.9 (±4.3)	3.9	12.8–40	10.7 (±3.3)	11.7	26.3–23.4	26.7 (±6.0)	28.2	9.7–39.7
Max Temperature of Warmest Month (° C)	20 (±4.2)	19.8	10–33	31.9 (±2.1)	32.2	26.2–36	28 (±2.0)	28.0	20.4–40
Min Temperature of Coldest Month (° C)	85.8 (±36)	88	-2.5–17.7	16.4 (±3.0)	16.2	10.7–22.9	9.6 (±2.4)	9.5	20.4–33.3
Temperature Annual Range (° C)	11.4 (±2.8)	11	8.5–26.6	15.6 (±2.7)	15.8	9–22.6	18.4 (±2.1)	18.6	10.4–24.5
Mean Temperature of Wettest Quarter (° C)	14.3 (±3.6)	14.3	5.4–25.1	24.6 (±1.6)	24.7	19.1–27.8	20.7 (±3.0)	21.4	13–26.8
Mean Temperature of Driest Quarter (° C)	14.0 (±3.7)	14.0	5.1–24.3	23.5 (±2.6)	23.4	18–28.3	16.6 (±2.7)	16.2	9.9–26.1
Mean Temperature of Warmest Quarter (° C)	14.8 (±3.8)	14.8	5.5–25.9	25.4 (±1.8)	25.4	20.2–29	22.2 (±1.9)	22.3	15.5–27.4
Mean Temperature of Coldest Quarter (° C)	13.6 (±3.6)	13.7	4.8–24	22.8 (±24)	22.5	17.3–27	15.5 (±2.2)	15.3	9.9–22.6
Annual Precipitation (mm)	2312 (±871)	2408	444–5881	1050 (±435)	999	385–3530	1528 (±251)	1476	1071–3343

Precipitation of Wettest Month (mm)	326 (±132)	323	81–725	215 (±73.3)	222	76–551	207 (±54)	196	128–394
Precipitation of Driest Month (mm)	75 (±419)	67	1–244	8.4 (±17)	3	0–198	69 (±39)	75	7–152
Precipitation Seasonality (mm)	43.4 (±159)	39	16–109	85 (±18)	87	23–119	37.5 (±25.8)	27	6–87
Precipitation of Wettest Quarter (mm)	856 (±350)	834	216–1958	563 (±199)	572	202–1393	568 (±144)	531	345–1163
Precipitation of Driest Quarter (mm)	266 (±142)	243	10–813	32 (±57)	13	1–644	234 (±126)	253	24–531
Precipitation of Warmest Quarter (mm)	617 (±248)	591	135–1490	217 (±107)	221	20–738	520 (±130)	494	251–1161
Precipitation of Coldest Quarter (mm)	497 (±289)	509	10–1334	152 (±237)	47	2–1195	249 (±131)	264	35–536
Sample size	494			351			594		

Table S5. Climate types of the data points of the tiger–cat species complex (in parenthesis the types of Köppen system).

Climate	Percentage of records (%)		
	tigrinus	***guttulus***	***pardinoides***
Tropical humid (Af. Am)	4.71	3.44	7.12
Tropical dry/savanna (Aw)	82.49	7.09	1.42
Arid (BSh)	12.8		
Sub–tropical hot (Cwa. Cfa)		62.35	0.57
Sub–tropical mild (Cwb. Cfb)		27.12	81.2
Temperate cold summer alpine (Cfc. ET)			9.69

Table S6. Description and source of environmental variables used for modeling the distribution of the tiger-cat species complex.

Code	Variable	Resolution	Year	Reference
ELEV	Mean Altitude above Sea Level	30 arcsec	2018	[26]
TRI	Terrain Ruggedness	30 arcsec	2018	[26]
NPP	Mean Net Primary Productivity	30 arcsec	1984 – 2014	[27]
GPP	Gross Primary Productivity	30 arcsec	1984 – 2014	[27]
HEIGHT	Mean Canopy Height	30 arcsec	2020	[28]
TC	Percent Tree Cover	30 arcsec	2021	[29]
BIO1	Annual Mean Temperature	30 arcsec	1970 – 2000	[30]
BIO2	Mean Diurnal Range	30 arcsec	1970 – 2000	[30]
BIO3	Isothermality	30 arcsec	1970 – 2000	[30]
BIO4	Temperature Seasonality	30 arcsec	1970 – 2000	[30]
BIO5	Max. Temperature of the Warmest Month	30 arcsec	1970 – 2000	[30]
BIO6	Min. Temperature of the Coldest Month	30 arcsec	1970 – 2000	[30]
BIO7	Annual Temperature Range	30 arcsec	1970 – 2000	[30]
BIO8	Mean Temperature of the Wettest Quarter	30 arcsec	1970 – 2000	[30]
BIO9	Mean Temperature of the Driest Quarter	30 arcsec	1970 – 2000	[30]
BIO10	Mean Temperature of the Warmest Quarter	30 arcsec	1970 – 2000	[30]
BIO11	Mean Temperature of the Coldest Quarter	30 arcsec	1970 – 2000	[30]
BIO12	Annual Precipitation	30 arcsec	1970 – 2000	[30]
BIO13	Precipitation of the Wettest Month	30 arcsec	1970 – 2000	[30]
BIO14	Precipitation of the Driest Month	30 arcsec	1970 – 2000	[30]
BIO15	Precipitation Seasonality	30 arcsec	1970 – 2000	[30]
BIO16	Precipitation of the Wettest Quarter	30 arcsec	1970 – 2000	[30]
BIO17	Precipitation of the Driest Quarter	30 arcsec	1970 – 2000	[30]
BIO18	Precipitation of the Warmest Quarter	30 arcsec	1970 – 2000	[30]
BIO19	Precipitation of the Coldest Quarter	30 arcsec	1970 – 2000	[30]
ECO	Ecoregion	30 arcsec	2017	[31]
LAND	Land cover	1/3 arcsec *	2021	[32]

Table S7. Reclassification table of the land cover layer used for modeling the distribution of the tiger-cat species complex.

Original value	Original label	New value	New label
10	Tree cover	1	Tree cover
20	Shrubland	2	Shrubland
30	Grassland	3	Unsuitable
40	Cropland	3	Unsuitable
50	Built–up	3	Unsuitable
60	Sparse vegetation	3	Unsuitable
70	Snow and ice	3	Unsuitable
80	Permanent water body	3	Unsuitable
90	Herbaceous wetland	3	Unsuitable
95	Mangrove	3	Unsuitable
100	Moss and lichen	3	Unsuitable

References:

1. Gray, J. E. Notes on certain species of cats in the collection of the British Museum. *Proc. Zool. Soc. London* (1867).
2. Kitchener, A. C. *et al.* A revised taxonomy of the Felidae. The final report of the Cat Classification Task Force of the IUCN/SSC Cat Specialist Group. *Cat News Spec. Issue* 80 (2017).
3. Allen, J. A. Notes on the synonymy and nomenclature of the smaller spotted cats of Tropical America. *Bull. Am. Museum Nat. Hist.* 341–419 (1919).
4. Hensel, R. F. Beiträge zur Kenntniss der Säugethiere Süd- Brasiliens. *Abhandlungen der Königlich Preuss. Akad. der Wiss.* 1–130 (1872).
5. Schreber, J. C. D. von. Die Säugethiere in Abbildungen nach der Natur mit Beschreibungen. (1775).
6. Trigo, T. C. *et al.* Molecular Data Reveal Complex Hybridization and a Cryptic Species of Neotropical Wild Cat. *Curr. Biol.* **23**, 2528–2533 (2013).
7. Tortato, F. R., Devlin, A. L., Boulhosa, R. L. P. & Hoogesteijn, R. Relative rarity of small wild cats in the Brazilian Pantanal. *Mammalia* **85**, 47–51 (2021).
8. Sunquist, M. & Sunquist, F. *Wild Cats of the World*. (2002).
9. Bonilla-Sánchez, A. Ecological niche modelling of the Leopardus tigrinus species complex (Mammalia, Felidae) sheds light on its elusive evolutionary history. (Pontifícia Universidade Católica do Rio Grande do Sul, Porto Alegre, Brazil, 2021).
10. Eisenberg, J. F. & Redford, K. H. *Mammals of the Neotropics (Volume 3): The Central Neotropics: Ecuador, Peru, Bolivia, Brazil*. (University of Chicago Press, 2000).
11. Oliveira, T. G. & Cassaro, K. *Guia de Campo dos Felinos do Brasil.* (Instituto Pró-Carnívoros/Fundação Parque Zoológico de São Paulo/SZB/Pró-Vida Brasil, 2005).
12. Breton, G. & Sanderson, J. Mistaken identity. *CAT News* 36–37 (2011).
13. de Oliveira, T. G., Fox-Rosales, L. A., Paemelaere, E. A. D. & Ferraz, K. M. P. M. de B. The dominant mesopredator and savanna formations shape the distribution of the threatened northern tiger cat (Leopardus tigrinus) in the Amazon. *Sci. Rep.* doi:10.138/s41598-022-21412-z.

14. Oliveira, T. G. *Neotropical cats: ecology and conservation*. (EDUFMA, 1994).
15. Husson, A. M. *The mammals of Suriname (Zoologische monographieen van het Rijksmuseum van Natuurlijke Historie ; no. 2)*. (Brill, 1978).
16. do Nascimento, F. O. & Feijó, A. Taxonomic revision of the tigrina Leopardus tigrinus (Schreber, 1775) species group (carnivora, felidae). *Pap. Avulsos Zool.* **57**, 231–264 (2017).
17. Lescroart, J. *et al.* The Neotropical cat genus Leopardus : a genomic history of rapid speciation and hybridization. in *101st Annual Meeting of the American Society of Mammalogists* (2022). doi:10.13140/RG.2.2.10252.80008.
18. de Oliveira, T. G., Fox-Rosales, L. A., Paemelaere, E. A. D. & Ferraz, K. M. P. M. de B. The dominant mesopredator and savanna formations shape the distribution of the rare northern tiger cat (Leopardus tigrinus) in the Amazon. *Sci. Rep.* 1–14 (2022) doi:10.1038/s41598-022-21412-z.
19. Cabrera, A. Catálogo de los mamíferos de América del Sur. *Rev. del Mus. Argentino Ciencias Nat. Bernardino Ridavadia Zool.* 1–370 (1958).
20. Wozencraft, W. C. Order Carnivora. in *Mammal Species of the World: A Taxonomic and Geographic Reference* (eds. Wilson, D. E. & Reeder, D. M.) 532–628 (John Hopkins University Press, 2005).
21. Ewer, R. F. *The Carnivores*. (Cornell University Press, 1973).
22. Nascimento, F. O. Do. Revisão taxonômica do gênero Leopardus Gray, 1842 (Carnivora, Felidae). (Universidade de São Paulo, 2010).
23. Eizirik, E. *et al.* Molecular genetics and evolution of melanism in the cat family. *Curr. Biol.* **13**, 448–453 (2003).
24. da Silva, L. G. *et al.* Biogeography of polymorphic phenotypes: Mapping and ecological modelling of coat colour variants in an elusive Neotropical cat, the jaguarundi (Puma yagouaroundi). *J. Zool.* **299**, 295–303 (2016).
25. Aximoff, I. *et al.* Melanism in the Brazilian pampas cat and range extension in the Atlantic Forest, Brazil. *Cat News* **74**, 29–32 (2021).
26. USGS. USGS EROS Archive - Digital Elevation - Shuttle Radar Topography Mission (SRTM) 1 Arc-Second Global. https://www.usgs.gov/centers/eros/science/usgs-eros-archive-digital-elevation-shuttle-radar-topography-mission-srtm-1-arc?qt-science_center_objects=0#qt-science_center_objects (2018).
27. Zhao, M., Heinsch, F. A., Nemani, R. R. & Running, S. W. Improvements of the MODIS terrestrial gross and net primary production global data set. *Remote Sens. Environ.* **95**, 164–176 (2005).
28. Potapov, P. *et al.* Mapping global forest canopy height through integration of GEDI and Landsat data. *Remote Sens. Environ.* **253**, 112165 (2021).
29. Hansen, M. C. *et al.* High-Resolution Global Maps of 21st-Century Forest Cover Change. *Science (80-.).* **342**, 850–853 (2013).
30. Fick, S. E. & Hijmans, R. J. WorldClim 2: new 1-km spatial resolution climate surfaces for global land areas. *Int. J. Climatol.* **37**, 4302–4315 (2017).
31. Olson, D. M. *et al.* Terrestrial ecoregions of the world: A new map of life on Earth. *Bioscience* **51**, 933–938 (2001).
32. Zanaga, D. *et al.* ESA WorldCover 10 m 2021 v200. (2022) doi:https://doi.org/10.5281/zenodo.7254221.
